# Preferential enhancement of nitrate utilization in rice by endophytic *Burkholderia vietnamiensis* RS1

**DOI:** 10.3389/fpls.2026.1753845

**Published:** 2026-05-12

**Authors:** Rina Shinjo, Naoya Ogawa, Kentaro Takahama, Motohiko Kondo

**Affiliations:** 1 Graduate School of Bioagricultural Sciences, Nagoya University, Nagoya, Japan; 2 Technical Center, Nagoya University, Nagoya, Japan

**Keywords:** *Burkholderia vietnamiensis*, endophyte, nitrate, rice, root

## Abstract

*Burkholderia vietnamiensis* RS1 is an endophytic bacterium that promotes rice growth by enhancing nitrogen acquisition. Transcriptomic analysis in our previous study showed that RS1 inoculation induces genes involved in nitrate transport and amino acid metabolism. However, its direct effects on nitrate uptake and the associated physiological mechanisms remained unclear. In this study, we quantitatively demonstrated that RS1 preferentially promotes nitrate uptake even in the presence of both nitrate and ammonium by applying ^15^N-labeled ammonium nitrate. Metabolic analysis by LC-MS/MS revealed reduced levels of specific amino acids (e.g., Ala, Ser, and Pro) and organic acids (pyruvic and citric acids) in RS1-inoculated roots, suggesting accelerated amino acid assimilation supported by increased carbon skeleton supply through the TCA cycle. RT-qPCR analysis in rice roots further revealed that inoculation with RS1 upregulated genes involved in the ammonium and nitrate transport, nitrogen assimilation, and the TCA cycle. Field experiments comparing nitrate- and urea-based fertilization revealed that RS1 significantly promoted rice growth under nitrate fertilization, but not under urea fertilization. These findings indicate that RS1 facilitates selective nitrate uptake and assimilation, contributing to improved nitrogen use efficiency and biomass production in rice. This study provides new insights into the functional role of endophytic bacteria in modulating nitrogen metabolism and highlights the potential of RS1 as a bioinoculant for sustainable agriculture, particularly under nitrate-rich conditions.

## 1 Introduction

Nitrogen (N) is a critical macronutrient for plant growth and crop productivity. To meet the growing global demand for grain crops, agricultural systems now rely heavily on N fertilizers synthesized from non-renewable energy sources (Ladha et al., 1997). The majority of plant-available N exists in the inorganic forms, including nitrate (NO_3_^−^) and ammonium (NH_4_^+^). These inorganic N forms are transported across the root plasma membrane (PM) by different families of transporters (Miller and Cramer, 2005). Due to the energy cost associated with NO_3_^−^ reduction, NH_4_^+^ is generally recognized as a more energy-efficient N form for plant assimilation (Bloom et al., 1992). Nevertheless, only a limited number of species thrive when ammonium is supplied as the sole N source, including boreal conifers (Kronzucker et al., 1997), tea plants (Ruan et al., 2000), and rice (Kronzucker et al., 1998a).

Rice (*Oryza sativa* L.) is one of the most important staple crops worldwide. The supply of N in paddy fields is recognized as a major limitation toward increased rice productivity (Cassman et al., 1993). Rice paddy soil usually becomes anoxic almost immediately beneath the soil-water interface (Revsbech et al., 1999). The immediate surface of rice roots supplies oxygen (O_2_) through leakage from aerenchyma (Revsbech et al., 1999), and therefore the release of O_2_ contributes to the aerobic microbial processes in the rice rhizosphere, such as nitrification (Briones et al., 2002; Ishii et al., 2011). Since NO_3_^–^ is rapidly lost through denitrification in the anoxic paddy soil, it is generally assumed that rice plants take up little NO_3_^–^ compared with NH_4_^+^ (Wang et al., 1993; Kronzucker et al., 1998b). However, NO_3_^–^ uptake by rice may be far more important than previously thought. It has been estimated that as much as 40% of the total N taken up in irrigated rice is absorbed as NO_3_^–^ (Kronzucker et al., 2000; Kirk and Kronzucker, 2005). Although NH_4_^+^ is energetically advantageous, rice and many other crops show improved growth when NO_3_^–^ is supplied alongside NH_4_^+^ (Ota and Yamamoto, 1989; Kronzucker et al., 1997; Santamaria and Elia, 1997). Rice growth, yield, and N acquisition are enhanced significantly when both NH_4_^+^ and NO_3_^–^ sources are provided simultaneously when compared with growth on NH_4_^+^ alone in solution culture (Cox and Reisenauer, 1973; Ying-Hua et al., 2006; Youngdahl et al., 1982). Although the synergistic mechanism of nitrate and ammonium uptake remains unclear, their co-provision can substantially alter carbon fluxes, including organic acid production via pyruvate kinase and TCA cycle enzymes, oxaloacetate formation by PEP carboxylase, and CO_2_ emission (Hachiya and Sakakibara, 2017).

*Burkholderia vietnamiensis* is a bacterium commonly found in soil, rhizosphere, and plants (Gillis et al., 1995; Govindarajan et al., 2008; Tang et al., 2010). It has been proposed as a plant growth-promoting bacterium (PGPB), as its inoculation significantly enhanced biomass production in rice and sugarcane (Govindarajan et al., 2006, Govindarajan et al., 2008; Tran Van et al., 2000). Inoculation of rice with *B. vietnamiensis* TVV75 significantly increased several yield components, resulting in a 13–22% increase in final grain yield (Tran Van et al., 2000). In addition, *B. vietnamiensis* LMG10929 showed a high capacity to colonize rice roots, enabling the establishment of a stable association with rice plants (Wallner et al., 2022). Together, these reports suggest a close association between *B. vietnamiensis* and rice, as well as its potential to enhance rice growth and productivity.

We previously reported that endophytic strain *B. vietnamiensis* RS1, isolated from sweet potato (Shinjo et al., 2018), promoted N acquisition in rice and further its biomass by a combination of root morphological changes and enhanced N absorption (Shinjo et al., 2020). This strain was primarily located on the rice root surface, within the apoplastic spaces of epidermal cells and cortex (Shinjo et al., 2020). Although RS1 can fix atmospheric nitrogen (N_2_ fixation), its contribution to rice growth was minor based on acetylene reduction activity (ARA) in roots inoculated with *B. vietnamiensis* RS1 (Shinjo et al., 2020). RNA-seq analysis in rice roots revealed that *B. vietnamiensis* RS1 upregulated the expression of genes related to NO_3_^–^ uptake, biosynthesis and translocation of amino acid, suggesting the enhanced N acquisition by *B. vietnamiensis* RS1 (Shinjo et al., 2020). Based on these observations, we hypothesized that RS1 inoculation selectively enhances NO_3_^–^ uptake; however, the mechanisms underlying this enhancement and the links between NO_3_^–^ uptake and downstream N assimilation and transport remain unclear. To address this knowledge gap, we conducted an integrated and multifaceted analysis of N uptake, assimilation, transport, and associated carbon metabolism to infer the mechanisms underlying RS1-mediated enhancement of N acquisition in rice.

This study aimed to identify the N form preferentially taken up in response to *B. vietnamiensis* RS1 inoculation and to assess its impact on subsequent assimilation processes. To this end, rice seedlings were inoculated with *B. vietnamiensis* RS1 and supplied with two types of ¹^5^N-labeled ammonium nitrate (NH_4_NO_3_). Isotopic analysis was used to quantify the absorbed N form, while LC-MS/MS was employed to assess changes in amino acid assimilation and organic acid dynamics. In addition, RT−qPCR analysis was conducted to examine RS1−induced transcriptional changes in inorganic N transport, N assimilation, and the TCA cycle in rice roots. Finally, a paddy field experiment was conducted to examine the growth-promoting effects of *B. vietnamiensis* RS1 under different N fertilization (urea and nitrate).

## 2 Materials and methods

### 2.1 Quantification of absorbed ^15^NH_4_^+^ and ^15^NO_3_^–^ by IRMS

Absorption of NH_4_^+^ and NO_3_^–^ was evaluated using ^15^N-labeled ammonium nitrate (NH_4_NO_3_). Before sowing rice seeds, ^15^NH_4_NO_3_ or NH_4_^15^NO_3_ (6.37 Atom % ^15^N each) solution was applied with the rate of 0.3 N kg^-1^ soil (14.6 mg N plant^-1^). Uniformly germinated seeds (*O. sativa* L. ssp. *japonica* cv. Nipponbare) treated with fungicides (Sportac, Nissan Chemical Industries, Tokyo, Japan) were sown into the autoclaved soil (Kantonosan, Tochigi, Japan) with fertilizers (0.4 g P_2_O_5_ kg^-1^, 0.4 g K_2_O kg^-1^) in a seedling tray and grown in the temperature-controlled room with artificial light (28 °C, 16/8 h light/dark, 260 μmol m^−2^ s^−1^) for 15 days. The soil was autoclaved only once prior to the addition of fertilizers. The water level was kept in 1 cm below the soil surface. To prepare the inoculant, *B. vietnamiensis* RS1 was cultured in YM broth at 28 °C with shaking overnight, and the cells were harvested by centrifugation (4 °C, 4500 × g, 10 min). Two milliliters of bacterial solution (OD_600_ = 0.01, approximately 2×10^7^ cells mL^-1^) were added directly to each seed to avoid inhomogeneous diffusion of applied labeled N. Control plants were supplied with two milliliters of sterile water. Inoculation was performed two times at one-week intervals from the time of sowing. Fifteen days after sowing, eight plants per treatment were sampled and freeze-dried. Freeze-dried shoots and roots were ground into a fine powder using a TissueLyser II (Qiagen, Hilden, Germany). Herein, one replication consists of two plants to get a sufficient amount of plant material for performing all the following analyses. The total N and ^15^N Atom % were evaluated by an isotope ratio mass spectrometer (IRMS, DELTA plus Advantage, Thermo Fisher Scientific, Waltham, MA, USA) connected to an elemental analyzer (Flash 2000, Thermo Fisher Scientific).

### 2.2 Analysis of free NO_3_^–^, amino acids, and organic acids in rice by LC-MS/MS

Extraction of free NO_3_^–^ (quantified as HNO_3_ in LC-MS/MS), amino acids, and organic acids was performed following procedures based on Czaban et al. (2016). Approximately 1–2 mg of the freeze-dried root or shoot samples were mixed by vortexing with 1 ml of ice-cold chloroform: methanol:1xTE buffer (pH=8.0) (1:3:1 [v/v/v]) solution containing 250 nM norvaline. Then 200 microliters of ice-cold chloroform and 1xTE buffer (pH=8) were added, mixed by vortex, and centrifuged (14,000 rpm, 4 °C, 10 min). The aqueous phase (600–700 μl) was taken and filtered through a 0.2 μm PTFE syringe filter (ADVANTEC, Tokyo, Japan).

The concentrations and ^15^N Atom% of free NO_3_^–^ and amino acids in the extract in each sample were determined by LC-MS/MS (LC: Vanquish UHPLC System Binary Pump H [Thermo Fisher Scientific], MS: Orbitrap Exploris 240 [Thermo Fisher Scientific]). Amino acids (Alanine [Ala], Arginine [Arg], Asparagine [Asn], Aspartic acid [Asp], Glutamine [Gln], Glutamic acid [Glu], Glycine [Gly], Histidine [His], Leucine [Leu], Lysine [Lys], Methionine [Met], Phenylalanine [Phe], Proline [Pro], Serine [Ser], Threonine [Thr], Tryptophan [Trp], Tyrosine [Tyr], Valine [Val]) were determined with an Intrada Amino Acid (3 µm, 100 x 2 mm; Imtakt, Kyoto, Japan) maintained at 37 °C. The mobile phases consisted of solvent A (0.3% formic acid in acetonitrile) and solvent B (acetonitrile-100 mM ammonium formate = 1:4, v/v) with a flow rate of 0.6 mL min^–1^, and the injection volume was 5 µl. For the determination of nitric acid (HNO_3_) and organic acids (pyruvic acid, oxaloacetic acid, citric acid, trans-aconitic acid, isocitric acid, α-ketoglutarate (α-KG), succinic acid, fumaric acid, malic acid), an Intrada Organic Acid column (3 μm, 100 x 2 mm; Imtakt, Kyoto, Japan) was maintained at 37 °C. The mobile phases consisted of solvent A (cetonitrile/water/formic acid = 10/90/0.1) and solvent B (acetonitrile-100 mM ammonium formate = 1:9, v/v) with a flow rate 0.2 mL min^–1^, and the injection volume was 5 µl. For MS detection, Orbitrap Exploris 240 mass spectrometer equipped with a heat electrospray ionization (H-ESI) source, operated in positive mode for the quantification of amino acids and negative mode for the quantification of nitric acid and organic acids. The spray voltage was set to 3.5 kV for amino acids and 2.5 kV for organic acids. The ion transfer tube temperature was set to 350 °C for amino acids and 320 °C for organic acids, while the vaporizer temperature was 350°C and 275 °C, respectively. Full-scan data were acquired at Orbitrap resolution of 60,000, over an m/z range of 60–600 for amino acids or 60–300 for organic acids.

### 2.3 RNA extraction, cDNA preparation, and RT-qPCR

The rest of rice seedlings described in 2.1 were grown until 21 DAS and used for gene expression analysis. Six plants exhibiting moderate growth were selected for each treatment, and two plants were combined to form one biological replicate. The samples were homogenized using a mortar and pestle under liquid nitrogen. Three biological replicates were prepared for each treatment.

Total RNA was extracted from homogenized rice root using a combination of the following kits: Fruit-mate for RNA purification (TaKaRa, Otsu, Japan), RNAiso Plus (TaKaRa), and NucleoSpin RNA Plant and Fungi (Macherey-Nagel, Düren, Germany). Approximately 400–700 mg of fresh weight of homogenized rice tissue was pretreated with Fruit-mate for RNA purification and centrifuged, following Matsunami et al. (2018). The supernatant was then treated with RNAiso Plus and further purified using the NucleoSpin RNA Plant and Fungi kit. Further details of the RNA extraction procedure are described in Matsunami et al. (2018). One microgram of total RNA was used to synthesize cDNA using PrimeScript RT reagent Kit (TaKaRa). RT-qPCR assay was performed with TB Green Premix Ex Taq (TaKaRa) on a Thermal Cycler Dice Real Time System (TaKaRa) according to the manufacturer’s instructions. The relative quantification of gene expression was determined using rice Ubiquitin1 expression as an internal reference.

Expression of genes encoding ammonium transporter (*OsAMT1;1*), nitratetransporter (*OsNRT1.1B [OsNPF6.5]*), glutamine synthetase (*OsGS1.1*, *OsGS1.2)*, glutamate synthase (*OsFd-GOGAT*), nitrate reductase (*OsNIA1*), and nitrite reductase (*OsNIR1*), isocitrate dehydrogenase (*ICDHc*), malic enzyme (*ME*), citrate synthase (*CS*), α-ketoglutarate dehydrogenase (*α-KGDH*), amino acid permeases (*OsAAP6*, *OsAAP7), and* cationic amino acid transporter (*OsCAT11)* was examined. The primers used for RT-qPCR are listed in **Supplementary Table 1** (Duan et al., 2007; Hu et al., 2015; Yun et al., 2008; Wang et al., 2018; Lee et al., 2020; Lian et al., 2021; Peng et al., 2014; Jin et al., 2024).

### 2.4 Evaluation of the effects of *B. vietnamiensis* RS1 on rice grown in the field

A field experiment was conducted in 2021 at the experimental field of Nagoya University, Togo, Aichi, Japan (latitude 35°6′N, longitude 137°5′E), with sandy clay soil classified as Typic Dystrudept. The soil chemical properties shown in **Supplementary Table 2** were measured by a commercial service (Katakura and Co-op Agri Corporation, Tokyo, Japan). The weather data for the experimental field is shown in **Supplementary Table 3** and was obtained from the nearest weather station of Japan Meteorological Agency (2021), located in Nagoya city. Uniformly germinated seeds (cv. Nipponbare) treated with fungicides (Sportac, Nissan Chemical Industries) were sown into the commercial nursery soil (Kantonosan) containing fertilizers (0.3 g N kg^–1^, 0.9 g P_2_O_5_ kg^–1^, 0.5 g K_2_O kg^–1^). Seedlings were grown for 29 days in a glasshouse in seedling trays (288 plants per tray) with submerged water at 2 cm above the soil surface. For inoculation, 500 mL of the bacterial cell suspension (OD_600_ = 0.001, 2 × 10^6^ cells mL^–1^) per seedling tray was carefully added to the submerged water. Uninoculated control plants were supplied with the same volume of sterile water. Bacterial inoculation was carried out four times in total before transplanting.

Control and inoculated seedlings were transplanted into the paddy field on May 25th, 2021, at a spacing of 30 cm × 15 cm. One hill consisted of one plant. Four replicated plots (1.2 m × 2.1 m) for each treatment were deployed with a randomized complete block design within a single field divided into two sections. Nitrogen was supplied to each plot individually prior to transplanting as controlled-release urea (LP40:S100:S140 = 1:1:1) (JCAM AGRI, Tokyo, Japan) (90 kg N ha^-1^) in the urea-plot and controlled-release nitrate (LS40:LS100:LS140 = 1:1:1) (JCAM AGRI) (90 kg N ha^-1^) in the nitrate-plot. Before N addition, phosphorus (90 kg P_2_O_5_ ha^-1^) and potassium (90 kg K_2_O ha^-1^) as compound PK fertilizer were applied for all treatments. The paddy fields were continuously flooded until harvesting. The heading date was on September 24th, 2021. At maturity (122 days after transplanting [DAT]), seven hills were harvested in each plot to determine the panicle numbers and the yields of brown rice and straw on an area basis. To evaluate the dry weight and N concentration, aboveground parts were oven-dried at 80 °C for 48 h and ground into fine powder using a Wonder Blender (Osaka Chemical Co., Osaka, Japan). The N content in the straw was determined by 2400 Series II CHNS/O Elemental Analyzer (Perkin Elmer, Tokyo, Japan).

### 2.5 Statistical analysis

In the seedling experiment, IRMS measurements were performed with four biological replicates. For LC-MS/MS analysis of ^15^N-labeled amino acids and free ^15^NO_3_^–^ was conducted with three replicates for statistical evaluation. Since no significant differences were found in dry weight, total N content, non-labeled amino acid concentrations, and organic acid concentrations between plants treated with ^15^NH_4_NO_3_ and NH_4_^15^NO_3_, these datasets were pooled. Consequently, statistical analyses were performed using either eight replicates (for dry weight and total N content) or six replicates (for non-labeled amino acids and organic acids). Differences between *B. vietnamiensis* RS1-inoculated and non-inoculated plants were assessed using Student’s *t*-test, with a significance threshold of *p* < 0.05 unless otherwise specified.

For the field experiment, statistical analyses were conducted separately for each N fertilization condition. Differences between RS1-inoculated and uninoculated plots within each N condition were evaluated using linear mixed-effects models with block included as a random effect (*p* < 0.05). All statistical analyses in this study were performed using JMP Student Edition 18.2.2 (SAS Institute Inc., Cary, NC, USA).

## 3 Results

### 3.1 Amount of absorbed ^15^NH_4_^+^ and ^15^NO_3_^–^ in rice seedlings

**Figure 1:**
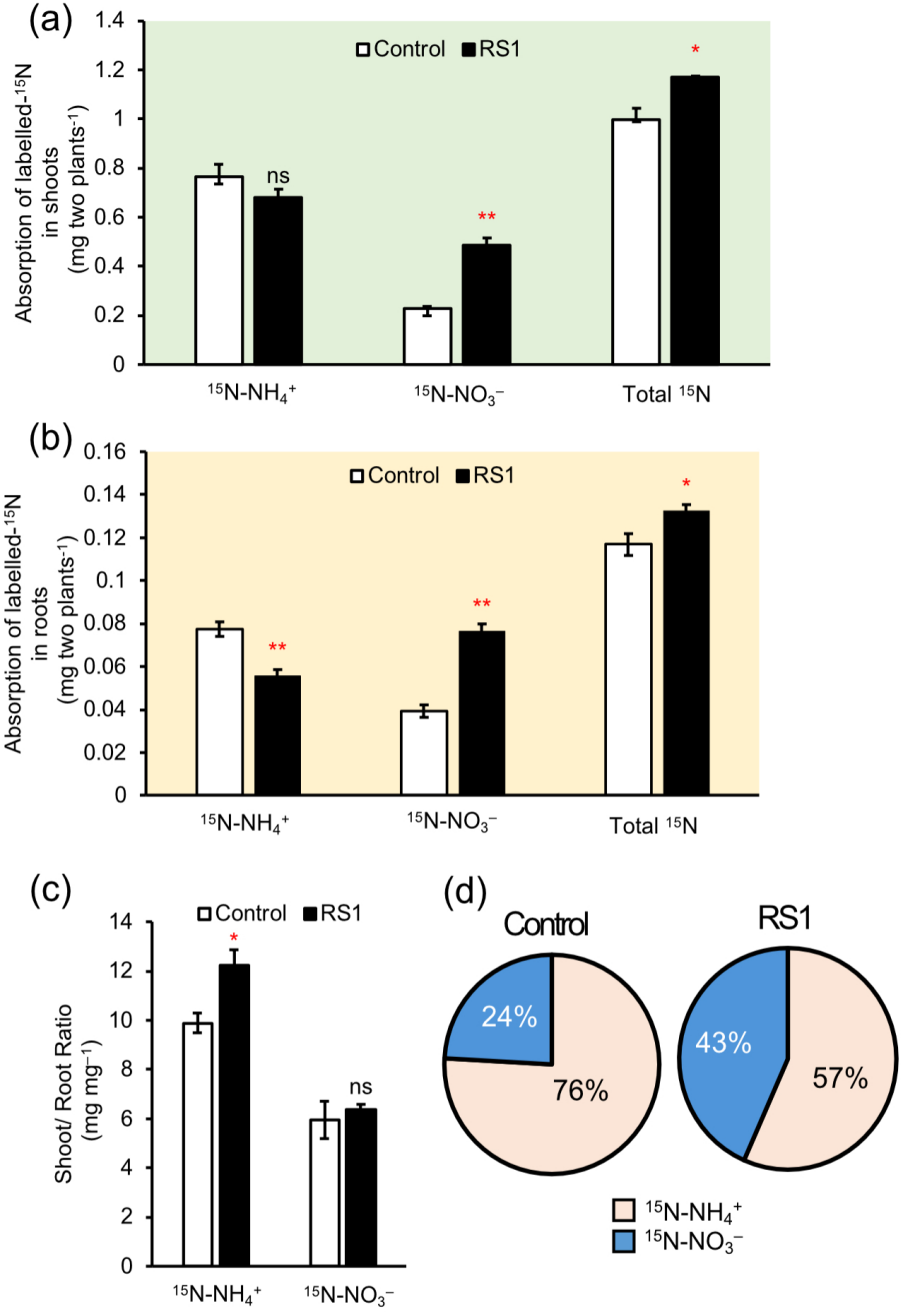
**(a)** Absorption of labeled ^15^N (^15^NH_4_^+, 15^NO_3_^–^) in shoots **(a)** and roots **(b)** inoculated with *B. vietnamiensis* RS1 (RS1) and uninoculated rice seedlings (Control) at 15 days after sowing (DAS). **(c)** Shoot/Root ratio of absorbed ^15^N (^15^NH_4_^+, 15^NO_3_^–^). **(d)** Percentage of the absorbed N sources (^15^NH_4_^+, 15^NO_3_^–^) in RS1 and Control. Error bars indicate the standard error (n = 4). Asterisks indicate significant differences (*p < 0.05, **p < 0.01, Student *t*-test). ns indicates no significant difference RS1 and Control.

The absorption of NH_4_^+^ and NO_3_^–^ in rice seedlings at 15 days after sowing (DAS) was independently investigated using ^15^N-labeled NH_4_NO_3_. **Figure 1** shows the amount and ratio of ^15^NH_4_^+^ and ^15^NO_3_^–^. There was no significant difference (*p* = 0.18) in ^15^NH_4_^+^ absorption in shoots between rice inoculated with *B. vietnamiensis* RS1 and control (**Figure 1a**). In contrast, ^15^NO_3_^–^ absorption in shoots was significantly higher in rice inoculated with RS1 than in control (**Figure 1a**). The total amount of absorbed ^15^N in shoots was also significantly increased in rice inoculated with *B. vietnamiensis* RS1 (**Figure 1a**). Similarly, absorption of ^15^NO_3_^–^ in root was also significantly increased, while those of ^15^NH_4_^+^ was decreased (**Figure 1b**). The shoot/root ratio of absorbed ^15^NH_4_^+^ was increased in rice inoculated with *B. vietnamiensis* RS1, while those of ^15^NO_3_^–^ was not changed (**Figure 1c**). The ratio of ^15^NO_3_^–^ in total absorbed ^15^N was higher in rice inoculated with *B. vietnamiensis* RS1 (43%) compared with control (24%) (**Figure 1d**). There were no differences in plant dry weight and total N content between the rice inoculated with *B. vietnamiensis* RS1 and control (**Supplementary Figures 1a, b**). Morphological changes in shoots and roots induced by inoculation were not visually observed (data not shown).

### 3.2 Concentrations of free ^15^NO_3_^–^, amino acids, and organic acids in rice seedlings

**Figure 2:**
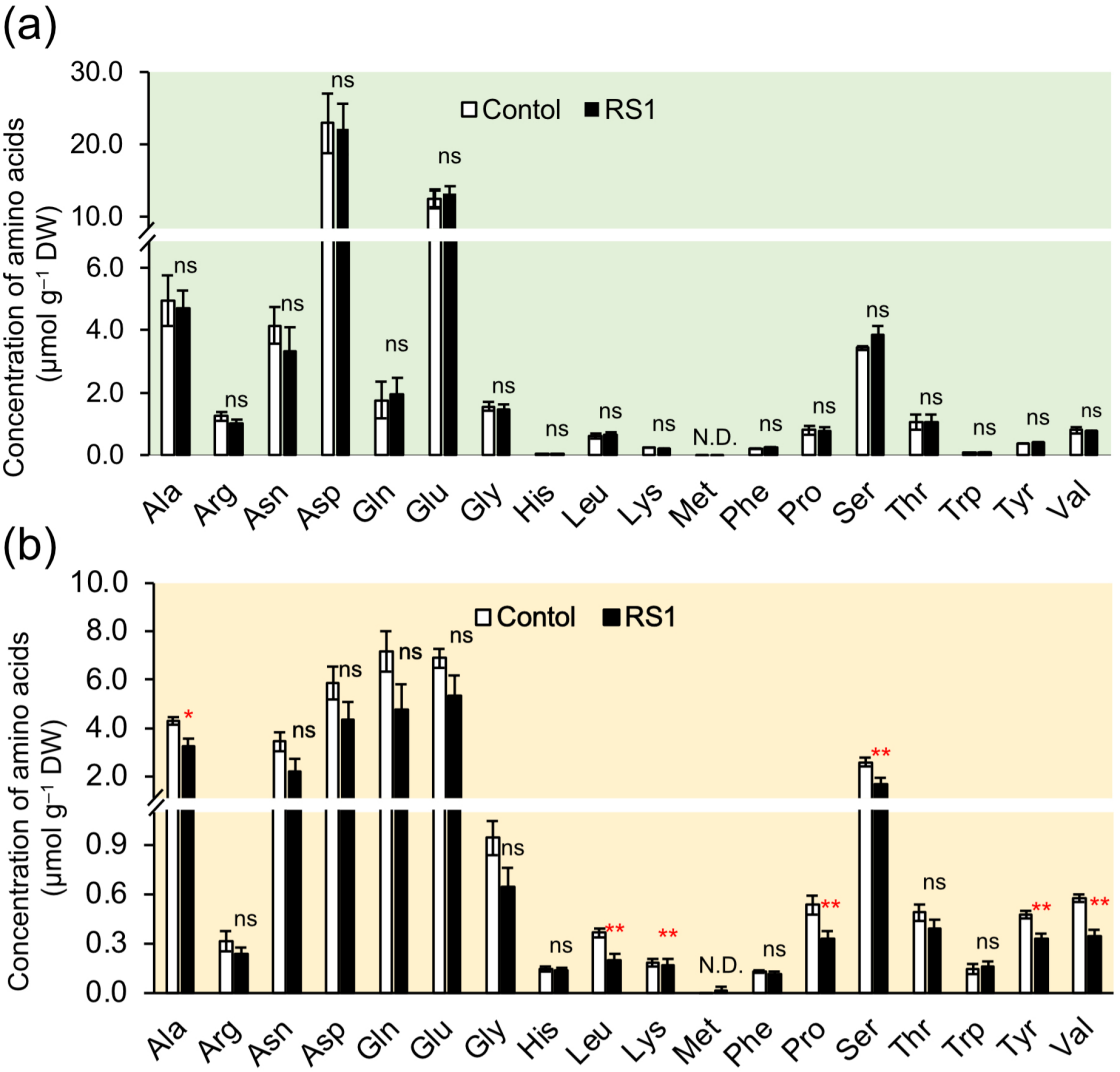
Non-labeled amino acids concentration in shoots **(a)** and roots **(b)** of rice seedlings inoculated with *B. vietnamiensis* RS1 (RS1) or without inoculation (Control) at 15 days after sowing (DAS). Error bars indicate the standard error (n = 6). Asterisks indicate significant differences (*p < 0.05, **p < 0.01; Student *t*-test), and ns indicates no significant difference RS1 and Control. (N) D., not detected.

**Figure 3:**
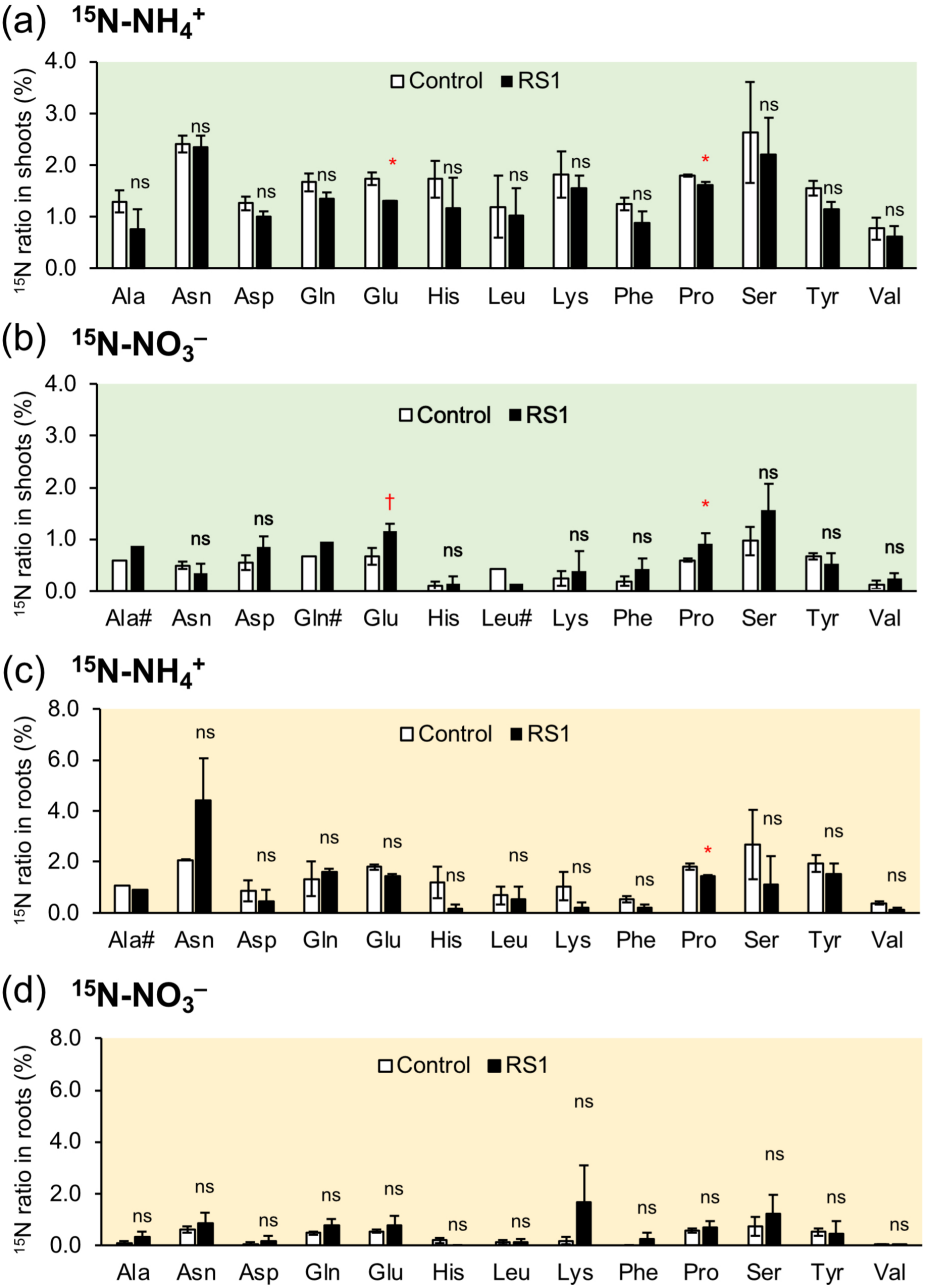
^15^N ratio of amino acids in shoots **(a, b)** and roots **(c, d)** of rice seedlings inoculated with *B. vietnamiensis* RS1 (RS1) or without inoculation (Control) at 15 days after sowing (DAS). **(a, c)** represent plants supplied with ^15^NH_4_^+^, while **(b, d)** represent plants supplied with ^15^NO_3_^–^. Error bars indicate the standard error (n = 3). Amino acids marked with a “#” were not statistically analyzed due to having only two replicates. Asterisks and daggers indicate significant differences (^†^*p* < 0.1, *p < 0.05; Student *t*-test), and ns indicates no significant difference RS1 and Control.

To examine the effect of *B. vietnamiensis* RS1 on rice N and carbon metabolism, the concentrations of free ^15^NO_3_^–^, amino acids (^15^N-labeled and non-labeled), and organic acids in rice seedlings inoculated with *B. vietnamiensis* RS1 were examined by LC-MS/MS analysis. In both shoots and roots, ^15^NO_3_^–^ concentration decreased slightly by inoculation, although the differences were not statistically significant (*p* = 0.31[shoot], 0.12[root]) (**Supplementary Figure 2**). Amino acid analysis revealed a significant reduction in the concentrations of alanine (Ala), leucine (Leu), lysine (Lys), proline (Pro), serine (Ser), tyrosine (Tyr), and valine (Val) in the roots inoculated with *B. vietnamiensis* RS1 (**Figure 2b**). Inoculation with *B. vietnamiensis* RS1 did not affect the amino acid concentration in the shoots (**Figure 2a**). **Figure 3** shows the results of isotopic analysis of the accumulation of ^15^NH_4_^+^ and ^15^NO_3_^–^ as amino acids. For some amino acids, particularly those indicated by the “#” symbol in **Figure 3**, ^15^N ratios were calculated from only two biological replicates because the ^15^N signal in one of the three analyzed samples was not detectable. In shoots inoculated with *B. vietnamiensis* RS1, the accumulation of ^15^NH_4_^+^ as amino acids tended to be decreased, with significant decreases in Glu and Pro (**Figure 3a**). Conversely, the accumulation of ^15^NO_3_^–^ as amino acids tended to be increased, with significant increases in Glu and Pro accumulation (**Figure 3c**). No significant differences were observed in either N form or amino acids in roots, but the trend was similar to that in shoots, except for Asn derived from ^15^NH_4_^+^ (**Figures 3b, d**).

**Figure 4:**
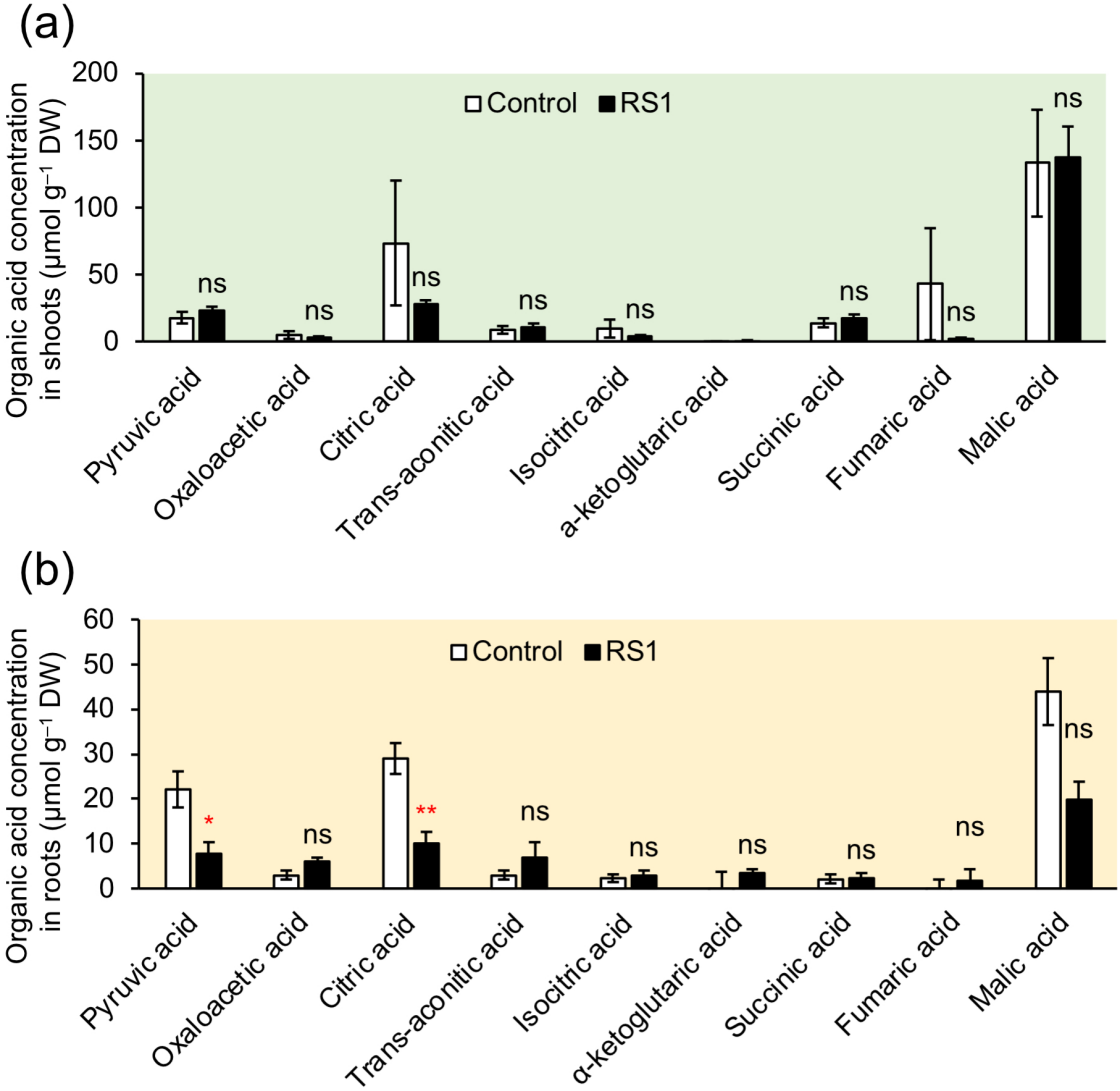
Organic acid concentration in shoot **(a)** and root **(b)** in rice seedlings inoculated with *B. vietnamiensis* RS1 (RS1) or without inoculation (Control) at 15 days after sowing (DAS). Error bars indicate the standard error (n = 6). Asterisks indicate significant differences (*p < 0.05, **p < 0.01; Student *t*-test), and ns indicates no significant difference RS1 and Control.

Among the organic acids analyzed, the concentrations of pyruvic acid and citric acid were significantly reduced in the roots inoculated with *B. vietnamiensis* RS1 (**Figure 4b**). No differences were observed in organic acid concentrations in the shoots between control and inoculated plants (**Figure 4a**).

### 3.3 Expression of genes related to N uptake and assimilation in rice

**Figure 5:**
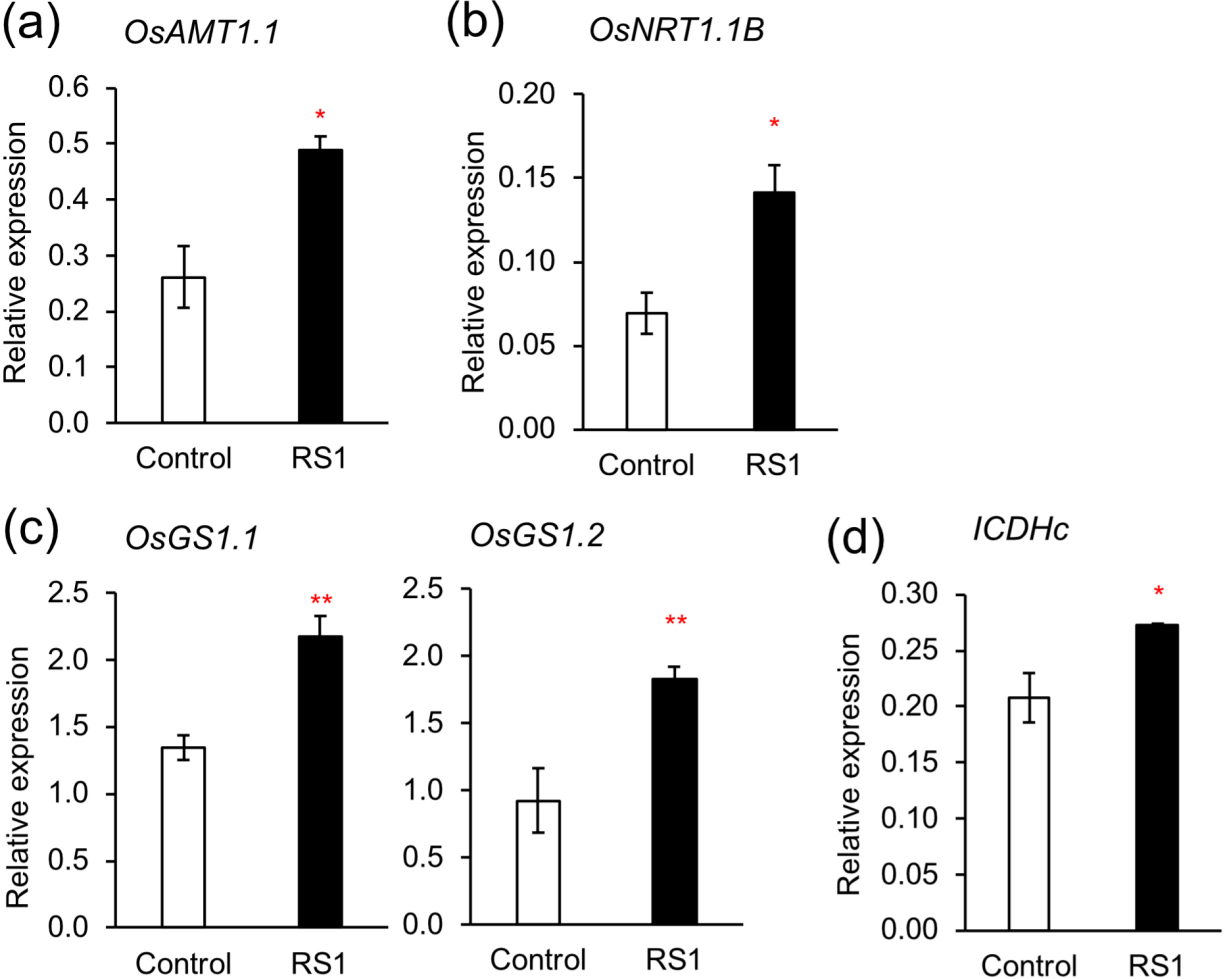
RT-qPCR-based expression analyses of genes in roots of Control and RS1 at 21 days after sowing (DAS). **(a)** Ammonium transporter *OsAMT1.1*, **(b)** nitrate transporter *OsNRT1.1B*, **(c)** glutamine synthetase *OsGS1.1* and *OsGS1.2*, **(d)** isocitrate dehydrogenase *ICDHc*. Error bars indicate the standard error (n = 3). Asterisks indicate significant differences between indicate significant differences between RS1 (rice seedlings inoculated with *B. vietnamiensis* RS1) and Control (rice seedlings without inoculation) (*p < 0.05, **p < 0.01, Student t-test).

To further investigate the effects of *B. vietnamiensis* RS1 on rice at the transcriptional level, the expression of genes related to N uptake and assimilation was analyzed in rice roots at 21 DAS. In roots inoculated with RS1, the expression of genes encoding ammonium transporter (*OsAMT1;1*) and nitrate transporter (*OsNRT1.1B*) was significantly upregulated compared with those in control (**Figures 5a, b**). Glutamine synthetase (*OsGS1.1*, *OsGS1.2)* was also significantly upregulated in RS1-inoculated roots (**Figure 5c**), while glutamate synthase (*OsFd-GOGAT*) was not affected by RS1 (**Supplementary Figure 3a**). Expression of genes responsible for nitrate reduction (*OsNIA1* and *OsNIR1*) was not changed (**Supplementary Figure 3b**). Isocitrate dehydrogenase (*ICDHc*), an enzyme involved in TCA cycle, was upregulated by RS1 (**Figure 5d**). However, expression of genes encoding other TCA enzymes, such as malic enzyme (*ME*), citrate synthase (*CS*), and α-ketoglutarate dehydrogenase (*α-KGDH*) was not significantly upregulated by RS1 (**Supplementary Figure 3c**). Expression of genes encoding amino acid transporters, including *OsAPL6* and *OsAPL7* (amino acid permeases), as well as *OsCAT11* (cationic amino acid transporter) was not affected by RS1 (**Supplementary Figure 3d**).

### 3.4 Effects of *B. vietnamiensis* RS1 on rice growth under different N fertilization in the field

The effects of different N forms on the effectiveness of *B. vietnamiensis* RS1 were examined in the field using the controlled-release urea/nitrate fertilizers. In the urea plot, there was no significant difference by inoculation with *B. vietnamiensis* RS1 (**Table 1**). On the other hand, inoculation with RS1 significantly increased the yields of brown rice and straw, and total dry mass in the nitrate plot (**Table 1**). The N content in the straw did not differ between the control and inoculated plants under either N fertilization (**Supplementary Figure 4**).

**Table 1:** Effect of *B. vietnamiensis* RS1 on rice yield with different N fertilization.

Form of fertilized nitrogen		Number of panicles per m^2^				Straw yield				Brown rice yield				Total dry biomass			
	Inoculation					g m^–2^				g m^–2^				g m^–2^			
Urea	Control	306.3	±	15.0	ns	790.1	±	68.5	ns	569.5	±	53.9	ns	1619.2	±	137.1	ns
	RS1	305.6	±	19.1		774.7	±	55.3		577.2	±	40.1		1601.4	±	111.7	
Nitrate	Control	192.9	±	9.9	ns	535.1	±	45.1	*	359.7	±	43.2	*	1087.5	±	96.9	*
	RS1	223.0	±	12.5		598.8	±	38.1		423.1	±	29.9		1283.4	±	71.9	

Values represent the mean ± standard error (n = 4). Asterisks indicate significant differences and ns indicates no significant difference between *B. vietnamiensis* RS1-inoculated and uninoculated plots within each N fertilization condition. Statistical significance was evaluated separately for each N fertilization condition using linear mixed−effects models with block treated as a random effect (*p < 0.05*).

## 4 Discussion

### 4.1 Enhanced nitrate uptake and amino acid biosynthesis in rice inoculated with *B. vietnamiensis* RS1

In this study, we provide quantitative evidence that NO_3_^–^ uptake is selectively stimulated in rice seedlings inoculated with endophytic bacterium *B. vietnamiensis* RS1 (**Figure 1**). When equal amounts of ¹^5^N-labeled NO_3_^-^ and NH_4_^+^ were supplied, *B. vietnamiensis* RS1 significantly increased the uptake of ^15^NO_3_^–^ in rice seedlings at 15 DAS (**Figures 1a, b**), raising the proportion of ^15^NO_3_^–^ in total absorbed ^15^N from 24% to 43% by inoculation (**Figure 1d**). Root N uptake is influenced by two primary factors: total root biomass and the N uptake efficiency per unit root mass. Since RS1 inoculation did not significantly alter root biomass at 15 DAS (**Supplementary Figure 1a**), the observed increase in ^15^NO_3_^–^ uptake likely reflects an enhancement in the uptake efficiency per unit root mass. This result suggests that RS1 may facilitate NO_3_^–^ acquisition, mainly by stimulating root physiological functions, including nitrate transporter activity, rather than by increasing root system development. Given that NO_3_^-^ acts not only as a nutrient but also as a signaling molecule (e.g., Hachiya and Sakakibara, 2017), the enhanced NO_3_^-^uptake induced by RS1 may have physiological effects beyond N acquisition itself, potentially influencing N assimilation and related metabolic processes.

To further investigate the impact of *B. vietnamiensis* RS1 on N assimilation following inorganic N uptake, LC-MS/MS analysis was conducted. Nitrate taken up into root cells is sequentially reduced to nitrite (NO_2_^-^) by nitrate reductase and assimilated into organic forms. In contrast, NH_4_^+^ can be directly incorporated into amino acids and other organic compounds, with its assimilation of NH_4_^+^ in root cells requiring carbon skeletons for amino acid synthesis via the glutamine synthetase (GS)/glutamate synthase (GOGAT) cycle. In the roots inoculated with RS1, the concentrations of certain amino acids and organic acids were decreased significantly (**Figure 2**, **4**). Notably, the reductions in pyruvic and citric acids may reflect changes in carbon metabolic flux, as these organic acids serve as key intermediates supplying carbon skeletons to the TCA cycle and downstream N assimilation pathways (Sweetlove et al., 2010). Despite increased ^15^NO_3_^–^ uptake (**Figure 1a**), the concentration of free nitrate (^15^NO_3_^–^) in root and shoot tended to be lower than those in control (**Supplementary Figure 2**). These results suggest that inoculation with *B. vietnamiensis* RS1 promoted biosynthesis of amino acids, resulting in the increased utilization of free NO_3_^–^ together with organic acids as carbon sources to support GS/GOGAT-mediated N assimilation.

In roots inoculated with RS1, the expression of nitrate transporter *OsNRT1.1B* was upregulated (**Figure 5b**), which was consistent with increased uptake of ^15^NO_3_^–^ in rice seedlings (**Figures 1a, b**). The expression of ammonium transporter *OsAMT1; 1* was also upregulated; however, no significant difference in ^15^NH_4_^+^ uptake was observed between treatments. Considering the remarkable upregulation of *OsGS1.1* and *OsGS1.2* by inoculation with *B. vietnamiensis* RS1, ammonium assimilation via GS appears to be enhanced in RS1-inoculated rice, which may in turn stimulate the transcriptional expression of *OsAMT1;1*. Glutamine synthesis requires α-KG as a carbon skeleton, which is supplied through the tricarboxylic acid (TCA) cycle. In rice inoculated with RS1, the expression of isocitrate dehydrogenase (*ICDHc*), which catalyzes the conversion of isocitrate to α−KG, was significantly upregulated (**Figure 5d**). This reaction links TCA cycle activity to N assimilation by supplying α-KG for the GS/GOGAT pathway (Foyer et al., 2011). Although the expression of other TCA cycle–related enzymes (*ME, CS, and α−KGDH*) did not differ significantly between RS1-inoculated and control plants, these genes showed a slight increasing trend (**Supplementary Figure 3c**). This trend suggests that a coordinated adjustment of carbon metabolism to support enhanced N assimilation (Masakapalli et al., 2013). Supporting this, isotopic analysis of amino acids revealed that nitrate was more actively incorporated into glutamate (Glu) and proline (Pro) in the shoots of RS1-inoculated rice compared to the control (**Figure 4a**), indicating enhanced GS/GOGAT pathway activity and subsequent Pro biosynthesis. The expression of genes involved in nitrate reduction, including *OsNIA* and *OsNIR*, was not altered by RS1 inoculation (**Supplementary Figure 3b**). Future studies are required to evaluate not only gene expression but also the enzymatic activities of these nitrate reduction–related enzymes to clarify whether their activities are affected by RS1 inoculation and to elucidate the underlying regulatory mechanisms.

**Figure 6:**
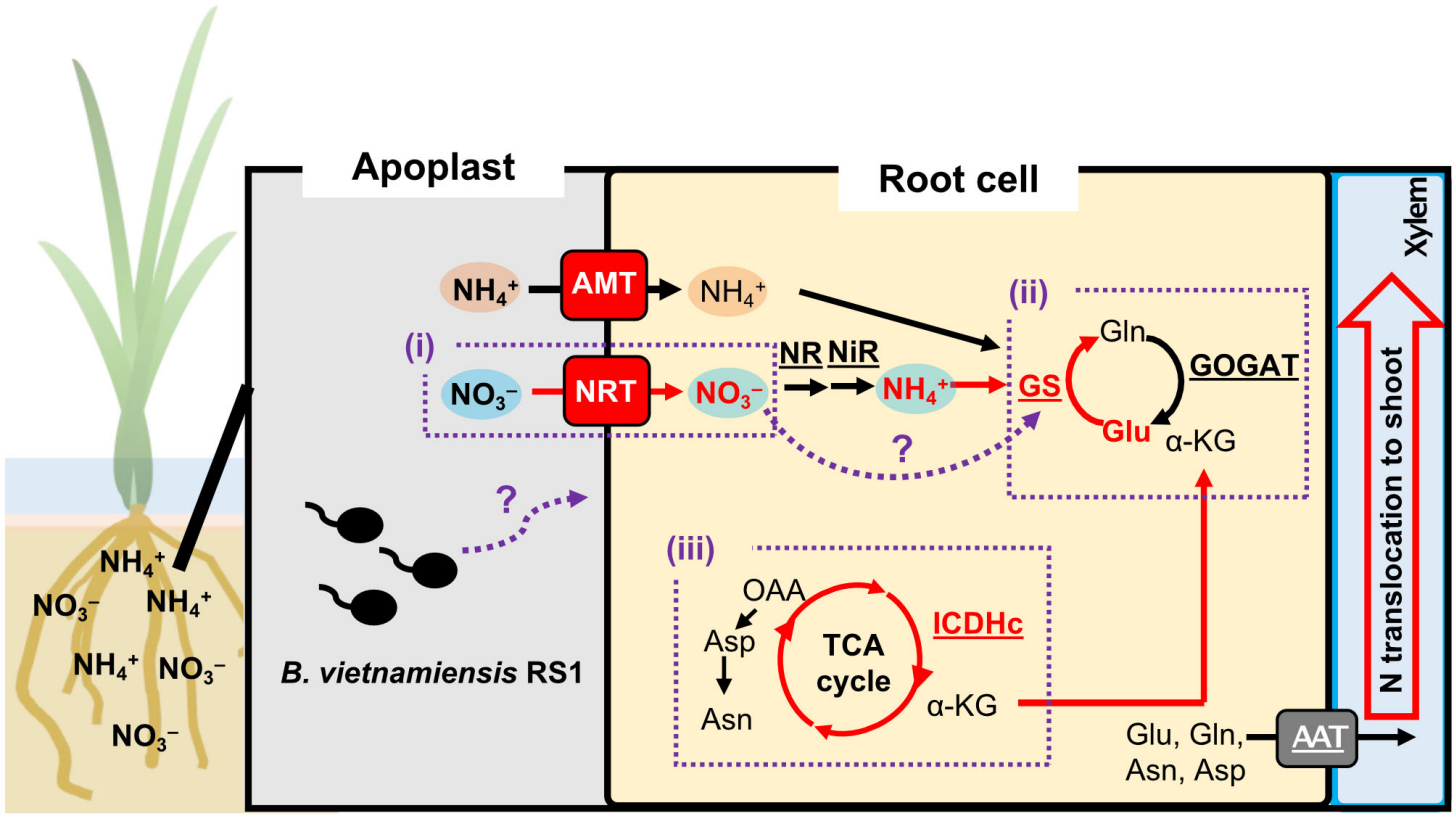
Hypothetical model of N uptake and assimilation in rice root cells mediated by *B. vietnamiensis* RS1. This figure illustrates a hypothetical model in which inoculation with *B. vietnamiensis* RS1 enhances NO_3_^-^ uptake in rice roots via NRT (i), accompanied by activation of the GS/GOGAT pathway for N assimilation (ii). In parallel, upregulation of ICDHc in the TCA cycle increases the supply of α−KG (iii), thereby strengthening the functional coupling between carbon metabolism and N assimilation and potentially promoting overall N assimilation in rice roots. Known pathways are shown with solid lines, whereas pathways activated by RS1 inoculation are indicated in red. Putative processes proposed in this study are indicated with purple dashed lines. NRT, nitrate transporters; AMT, ammonium transporters; AAT, amino acid transporters; NR, nitrate reductase; NiR, nitrite reductase; GS, glutamine synthetase; GOGAT, glutamate synthase; ICDHc, isocitrate dehydrogenase. Enzymes are underlined.

Inoculation with *B. vietnamiensis* RS1 affected N translocation from root to shoot. Most of the absorbed ¹^5^NO_3_^-^ and ¹^5^NH_4_^+^ was transported to the shoot from the root regardless of inoculation (**Figure 1a**). The shoot/root ratio of absorbed ¹^5^NH_4_^+^ was significantly higher in RS1-inoculated seedlings, whereas no significant difference was observed for ¹^5^NO_3_^-^ (**Figure 1c**). This suggests that RS1 inoculation simultaneously enhanced ¹^5^NO_3_^-^ uptake, assimilation, and translocation in a balanced manner, resulting in a consistent shoot/root ratio across treatments (**Figure 6**). In contrast, for NH_4_^+^, although RS1 inoculation did not affect the total uptake, it possibly promoted assimilation into amino acids and subsequent translocation to the shoot. This imbalance between uptake and post−uptake processes likely resulted in the increased shoot/root ratio observed for ^15^NH_4_^+^. To further examine whether RS1 inoculation transcriptionally affects amino acid transport, we analyzed the expression of amino acid transporter genes, including *OsAPL6* and *OsAPL7*, as well as *OsCAT11*. However, although the expression of these genes was detectable, none of these genes showed increased expression in RS1−inoculated roots (**Supplementary Figure 3d**), suggesting that the elevated shoot translocation of ¹^5^NH_4_^+^ was not driven by transcriptional activation of these amino−acid transporters.

### 4.2 Hypothetical mechanisms of RS1-mediated nitrate uptake enhancement

What mechanisms underlie RS1-mediated enhancement of nitrate uptake and subsequent assimilation? As mentioned above, RT-qPCR analysis showed that RS1 inoculation upregulated the expression of the nitrate transporters *OsNRT1.1B* (**Figure 5b**). *OsNRT1.1B* encodes a dual-affinity transporter and sensor for NO_3_^–^, thereby linking external NO_3_^–^ availability to downstream regulatory responses, including NO_3_^–^ assimilation and root-to-shoot N signaling (Plett et al., 2010; Ho et al., 2009; Liu et al., 1999). Previous research showed that, in the near-isogenic line (NIL) of *japonica* rice carrying the *indica*-type *OsNRT1.1B* allele, NO_3_^–^ uptake and root-to-shoot N transport were enhanced compared to lines carrying the *japonica*-type *OsNRT1.1B* allele (Hu et al., 2015). This pattern is consistent with the response observed in this study (**Figures 1a, b**), suggesting that the expression of *OsNRT1.1B*, along with NO_3_^–^ absorption and subsequent assimilation, may be upregulated by specific endophytic or rhizosphere bacteria such as RS1.

NO_3_^-^ transport is not solely dependent on transporter abundance—it also requires a proton motive force across the PM, which energizes H^+^-coupled transporters (Muratore et al., 2021). A major driving force for the transport of nitrate and other ions is the transmembrane electrochemical gradient maintained by PM H^+^-ATPase, whose activity is critical for nutrient acquisition (White, 2003). In some previous reports, PGPB enhanced nutrient uptake by influencing ion transport systems (Lin et al., 1983; Mantelin et al., 2006; Pii et al., 2015). For instance, inoculation of wheat seedlings with *Azospirillum brasilense* Cd increased root H^+^ efflux, likely via bacterial signaling molecules (Bashan et al., 1989; Bashan and Levanony, 1991). Similar effects were observed in oilseed-rape inoculated with *Achromobacter* sp., which enhanced NO_3_^-^ and K^+^ influx (Bertrand et al., 2000). These findings suggest that the possibility that stimulation of H^+^-ATPase activity and subsequent enhancement of NO_3_^-^ uptake may also occur in roots inoculated with *B. vietnamiensis* RS1. Although direct evidence is currently lacking, some microorganisms secrete bioactive compounds that modulate ion transport systems in plant roots. In *Trichoderma* spp., several secreted peptaibols have been shown to stimulate the PM H^+^-ATPase, thereby promoting plant growth (López-Coria et al., 2016; Bjørk et al., 2023). By analogy, it is conceivable that such microbe-derived molecules could enhance NRT-mediated NO_3_^-^ uptake, which may provide a plausible explanation for the increased NO_3_^-^ absorption observed in RS1-inoculated plants.

In addition to its role as a nutrient, NO_3_^-^ functions as a key signaling molecule in plants. It influences a wide range of physiological and developmental processes, including genome-wide gene expression, leaf expansion, root and seed morphology, seed dormancy, and the initiation of flowering (Hachiya and Sakakibara, 2017). In *Arabidopsis thaliana*, NO_3_^-^ acts as a N satiety signal, promoting the expression of nitrate assimilation genes in the shoots (Okamoto et al., 2019). It also facilitates lateral root development by enhancing auxin uptake via the *NRT1.1* transporter (Krouk et al., 2010). Moreover, NO_3_^-^ contributes to increased carbohydrate mobilization and stimulates glycolytic activity (Scheible et al., 1997; Sakakibara et al., 2006). Yun et al. (2008) showed that nitrate supply enhances GS activity as well as *OsGS1;1* expression in rice. In RS1-inoculated rice plants, NO_3_^-^ uptake was significantly increased (**Figures 1a, b**), suggesting that elevated NO_3_^-^ availability is one possible factor contributing to GS activation (**Figure 6**), while the underlying regulatory mechanism still needs to be clarified. These findings suggest that the enhanced NO_3_^-^ uptake observed in RS1-inoculated rice may have triggered downstream signaling pathways that regulate not only N assimilation but also the metabolic processes involved in organic acid supply, thereby highlighting the dual role of nitrate as both a nutrient and a signaling molecule.

### 4.3 Field performance of RS1 under different nitrogen sources

Previous studies have shown that PGPB enhance nutrient uptake and plant growth, leading to improved nutrient use efficiency (Backer et al., 2018; Ren et al., 2025). Growth-promoting effects of *B. vietnamiensis* on rice in the field have also been reported (Tran Van et al., 2000); however, the influence of soil N form on these effects remains unclear. In our field experiment, rice inoculated with *B. vietnamiensis* RS1 exhibited increased brown rice and straw yields, as well as total dry biomass, when controlled-release nitrate was used as a basal fertilizer (**Table 1**). In contrast, no growth differences were observed between inoculated and non-inoculated plants when controlled-release urea was used as a basal fertilizer. Although total dry biomass was lower in the controlled-release nitrate plots compared to the controlled-release urea plots (**Table 1**), the positive impact of RS1 inoculation on rice growth was more pronounced under nitrate fertilization. This reduction in biomass may be partly explained by greater N losses through denitrification in the reduced soil layer of nitrate-treated plots. Additionally, the high N availability generated through urea hydrolysis likely masked or diminished the detectable contribution of RS1 in the urea treatment, resulting in no significant differences between RS1−inoculated and control plants. These findings are consistent with the trends observed in seedling experiments in this study, where selective enhancement of nitrate uptake was detected in RS1-inoculated rice (**Figures 1a, b**). However, a previous study reported a tendency for improved growth of RS1-inoculated rice even under controlled-release urea fertilization (Shinjo et al., 2020), which appears to contradict the present results. Such discrepancies may be attributable to environmental factors influencing nitrification, including specific weather patterns and microbial community composition in paddy soils.

Alternate wetting and drying (AWD), a widely adopted water-saving irrigation method, creates more aerobic conditions than continuous flooding (Lampayan et al., 2015) and increased soil NO_3_^-^ level during the dry period via the nitrification process (Hmwe et al., 2022). Therefore, AWD has the potential to maximize the growth-promoting effects of *B. vietnamensis* RS1. The effect of RS1 may differ between the two subspecies of rice, *indica* and *japonica*. *Indica* cultivars generally possess a higher potential for NO_3_^–^ uptake than *japonica* cultivars (Chanh et al., 1981), and they also tend to exhibit greater rhizosphere oxidation capacity. For instance, the *indica* cultivar Yangdao 6 releases more oxygen into the rhizosphere than the *japonica* cultivar Nongken 57, thereby promoting higher nitrification activity. This results in higher NO_3_^–^ concentrations in the rhizosphere and consequently greater N uptake (Li et al., 2008). These findings suggest that the RS1-induced enhancement of NO_3_^–^ absorption may differ between *indica* and *japonica* cultivars and may be particularly pronounced in those with higher rhizosphere oxidation and NO_3_^–^ uptake capacities. Future studies should evaluate the effects of *B. vietnamiensis* RS1 under diverse field conditions. In addition, confirming the absence of pathogenicity in this strain is essential, as *B. vietnamiensis* also isolated from human lungs has been reported as an opportunistic pathogen (Govindarajan et al., 2008). Identifying RS1-derived compounds that activates nitrate transporters and exploring their application could be a promising approach.

In the present study, the *japonica* cultivar Nipponbare was employed as a model genotype to reduce genetic variability and to focus on mechanistic insights into microbial-driven N acquisition. However, to generalize the findings of this study, future validation using diverse rice cultivars will be required. Furthermore, assessing the agronomic relevance of the findings reported here will require field evaluations conducted under a range of environmental conditions and across multiple growing seasons. Given that *B. vietnamiensis* RS1 is phylogenetically related to members of the *Burkholderia cepacia* complex, which are known to cause severe pneumonia in individuals with cystic fibrosis, comprehensive biosafety and risk assessments will be required prior to any agricultural application. From this perspective, if comparable growth-promoting effects can be achieved using culture supernatants or inactivated bacterial cells rather than live cells, these alternatives could represent promising approaches for practical applications.

## 5 Conclusion

In this study, we provide quantitative evidence using ^15^N isotopic tracer that the endophytic bacterium *B. vietnamiensis* RS1 enhances preferential uptake and assimilation of NO_3_^-^ in rice seedlings. Gene expression analysis further confirmed that inoculation with RS1 promoted NO_3_^-^ uptake via nitrate transporter and enhanced its assimilation into amino acids. These responses are likely mediated by the activation of the GS/GOGAT pathway together with the coordinated regulation of carbon metabolism. Field experiments showed that RS1 inoculation significantly improved rice growth under controlled-release nitrate fertilization. Importantly, these results suggest that RS1 inoculation may contribute to more efficient utilization of applied N fertilizer, thereby supporting stable crop productivity with potentially reduced N input. Future research should elucidate the molecular mechanisms by which RS1 activates NO_3_^-^ uptake and N assimilation in rice and assess its effectiveness under diverse environmental conditions. Collectively, these findings highlight the potential of *B. vietnamiensis* RS1 as a sustainable strategy for improving N use efficiency in rice cultivation.

## Data Availability

The original contributions presented in the study are included in the article/**Supplementary Material**. Further inquiries can be directed to the corresponding authors.

## Supplementary material

Supplementary Figure 1 Dry weight **(a)** and N content **(b)** of rice seedlings inoculated with *B. vietnamiensis* RS1 (RS1) or without inoculation (Control) at 15 days after sowing (DAS). Error bars indicate the standard error (n = 8). ns indicates no significant difference RS1 and Control (*p < 0.05*, Student *t*-test).

Supplementary Figure 2 Concentration of labeled free ^15^NO_3_^–^ in rice seedlings supplied with ^15^NO_3_^–^ at 15 days after sowing (DAS). Error bars indicate the standard error (n = 4). ns indicates no significant difference RS1 and Control (*p < 0.05*, Student *t*-test).

Supplementary Figure 3 RT-qPCR-based expression analyses of genes in roots of Control and RS1 at 21 days after sowing (DAS). **(a)** glutamate synthase (*OsFd-GOGAT)*, **(b)** nitrate reductase (*OsNIA1*) and nitrite reductase (*OsNIR1*), **(c)** malic enzyme (*ME*), citrate synthase (*CS*), α-ketoglutarate dehydrogenase (*α-KGDH*), **(d)** amino acid transporters (*OsAAP6, OsAAP7, OsCAT11*). Error bars indicate the standard error (n = 3). ns indicates no significant difference RS1 and Control (*p < 0.05*, Student *t*-test).

Supplementary Figure 4 Nitrogen content in the rice straw. Error bars indicate the standard error (n = 4). ns indicates no significant difference between RS1 and Control within each N fertilization condition. Statistical significance was evaluated separately for each N fertilization condition using linear mixed−effects models with block treated as a random effect (*p < 0.05*).

Supplementary Table 1 Primers used in this study.

Supplementary Table 2 Physical and chemical properties of experimental paddy field soil.

Supplementary Table 3 Weather data at a site near the experimental paddy field during the experimental period in Nagoya, Aichi Prefecture, Japan.

## References

[B1] Backer R. Rokem J. S. Ilangumaran G. Lamont J. Praslickova D. Ricci E. . (2018). Plant growth-promoting rhizobacteria: context, mechanisms of action, and roadmap to commercialization of biostimulants for sustainable agriculture. Front. Plant Sci. 9, 1473. doi: 10.3389/fpls.2018.01473. PMID: 30405652 PMC6206271

[B2] Bashan Y. Levanony H. (1991). Alterations in membrane potential and in proton efflux in plant roots induced by Azospirillum brasilense. Plant Soil 137, 99–103. doi: 10.1007/BF02187439. PMID: 30311153

[B3] Bashan Y. Levanony H. Mitiku G. (1989). Changes in proton efflux of intact wheat roots induced by Azospirillum brasilense Cd. Can. J. Microbiol. 35, 691–697. doi: 10.1139/m89-113. PMID: 34819996

[B4] Bertrand H. Plassard C. Pinochet X. Touraine B. Normand P. Cleyet-Marel J. (2000). Stimulation of the ionic transport system in Brassica napus by a plant growth-promoting rhizobacterium (Achromobacter sp.). Can. J. Bot. 46, 229–236. doi: 10.1139/w99-137. PMID: 10749536

[B5] Bjørk P. K. Johansen N. T. Havshøi N. W. Rasmussen S. A. Ipsen J.Ø. Isbrandt T. . (2023). Trichoderma harzianum peptaibols stimulate plant plasma membrane H+-ATPase activity. ACS Omega. 8, 34928–34937. doi: 10.1021/acsomega.3c04299. PMID: 37779967 PMC10536087

[B6] Bloom A. J. Sukrapanna S. S. Warner R. L. (1992). Root respiration associated with ammonium and nitrate absorption and assimilation by barley. Plant Physiol. 99, 1294–1301. doi: 10.1104/pp.99.4.1294. PMID: 16669035 PMC1080623

[B7] Briones A. M. Okabe S. Umemiya Y. Ramsing N.-B. Reichardt W. Okuyama H. (2002). Influence of different cultivars on populations of ammonia-oxidizing bacteria in the root environment of rice. Appl. Environ. Microbiol. 68, 3067–3075. doi: 10.1128/AEM.68.6.3067-3075.2002. PMID: 12039768 PMC123923

[B8] Cassman K. Kropff M. Gaunt J. Peng S. (1993). Nitrogen use efficiency of rice reconsidered: What are the key constraints? Plant Soil 155, 359–362. doi: 10.1007/BF00025057. PMID: 30311153

[B9] Chanh T. T. Tsutsumi M. Kurihara K. (1981). Comparative study on the response of Indica and Japonica rice plants to ammonium and nitrate nitrogen. Soil Sci. Plant Nutr. 27, 83–92. doi: 10.1080/00380768.1981.10431257. PMID: 37339054

[B10] Cox W. J. Reisenauer H. (1973). Growth and ion uptake by wheat supplied nitrogen as nitrate, or ammonium, or both. Plant Soil 38, 363–370. doi: 10.1007/bf00779019. PMID: 30311153

[B11] Czaban W. Jämtgård S. Näsholm T. Rasmussen J. Nicolaisen M. H. Murphy D. V. (2016). Direct acquisition of organic N by white clover even in the presence of inorganic N. Plant Soil 407, 91–107. doi: 10.1007/s11104-016-2896-z. PMID: 30311153

[B12] Duan Y. H. Zhang Y. L. Ye L. T. Fan X. R. Xu G. H. Shen Q. R. (2007). Responses of rice cultivars with different nitrogen use efficiency to partial nitrate nutrition. Ann. Bot. 99, 1153–1160. doi: 10.1093/aob/mcm051. PMID: 17428833 PMC3244343

[B13] Foyer C. H. Noctor G. Hodges M. (2011). Respiration and nitrogen assimilation: targeting mitochondria-associated metabolism as a means to enhance nitrogen use efficiency. J. Exp. Bot. 62, 1467–1482. doi: 10.1093/jxb/erq453. PMID: 21282329

[B14] Gillis M. Van Van T. Bardin R. Goor M. Hebbar P. Willems A. . (1995). Polyphasic taxonomy in the genus Burkholderia leading to an emended description of the genus and proposition of Burkholderia Vietnamiensis sp. nov. for N2-fixing isolates from rice in Vietnam. Int. J. Syst. Bacteriol. 45, 274–289. doi: 10.1099/00207713-45-2-274. PMID: 27077644

[B15] Govindarajan M. Balandreau J. Kwon S. W. Weon H. Y. Lakshminarasimhan C. (2008). Effects of the inoculation of Burkholderia Vietnamiensis and related endophytic diazotrophic bacteria on grain yield of rice. Microb. Ecol. 55, 21–37. doi: 10.1007/s00248-007-9247-9. PMID: 17406771

[B16] Govindarajan M. Balandreau J. Muthukumarasamy R. Revathi G. Lakshminarasimhan C. (2006). Improved yield of micropropagated sugarcane following inoculation by endophytic Burkholderia Vietnamiensis. Plant Soil 280, 239–252. doi: 10.1007/s11104-005-3223-2. PMID: 30311153

[B17] Hachiya T. Sakakibara H. (2017). Interactions between nitrate and ammonium in their uptake, allocation, assimilation, and signaling in plants. J. Exp. Bot. 68, 2501–2512. doi: 10.1093/jxb/erw449. PMID: 28007951

[B18] Hmwe K. K. Yashima M. M. Inubushi K. (2022). Nitrogen transformation in paddy soil and its effect on rice as affected by different N sources and water regimes. Trop. Agric. Dev. 66, 1–11. doi: 10.11248/jsta.66.1

[B19] Ho C. H. Lin S. H. Hu H. C. Tsay Y. F. (2009). CHL1 functions as a nitrate sensor in plants. Cell. 138, 1184–1194. doi: 10.1016/j.cell.2009.07.004. PMID: 19766570

[B20] Hu B. Wang W. Ou S. Tang J. Li H. . (2015). Variation in *NRT1.1B* contributes to nitrateuse divergence between rice subspecies. Nat. Genet. 47, 834–838. doi: 10.1038/ng.3337 26053497

[B21] Ishii S. Ikeda S. Minamisawa K. Senoo K. (2011). Nitrogen cycling in rice paddy environments: past achievements and future challenges. Microbes Environ. 26, 282–292. doi: 10.1264/jsme2.me11293. PMID: 22008507

[B22] Japan Meteorological Agency (2021). Japan Meteorological Agency. Available online at: https://www.jma.go.jp/jma/index.html (Accessed March 27, 2026).

[B23] Jin F. Xie P. Li Z. Wu B. Huang W. Fang Z. (2024). Blocking of amino acid transporter OsAAP7 promoted tillering and yield by determining basic and neutral amino acids accumulation in rice. BMC Plant Biol. 24, 447. doi: 10.1186/s12870-024-05159-5. PMID: 38783192 PMC11112796

[B24] Kirk G. Kronzucker H. (2005). The potential for nitrification and nitrate uptake in the rhizosphere of wetland plants: a modelling study. Ann. Bot. 96, 639–646. doi: 10.1093/aob/mci216. PMID: 16024557 PMC4247031

[B25] Kronzucker H. J. Glass A. D. M. Siddiqi M. Y. Kirk G. J. D. (2000). Comparative kinetic analysis of ammonium and nitrate acquisition by tropical lowland rice: implications for rice cultivation and yield potential. New Phytol. 145, 471–476. doi: 10.1046/j.1469-8137.2000.00606.x. PMID: 33862905

[B26] Kronzucker H. J. Kirk G. J. Yaeesh Siddiqi M. Glass A. D. (1998a). Effects of hypoxia on 13NH4+ fluxes in rice roots: kinetics and compartmental analysis. Plant Physiol. 116, 581–587. doi: 10.1104/pp.116.2.581. PMID: 9490761 PMC35115

[B27] Kronzucker H. J. Schjoerring J. K. Erner Y. Kirk G. J. Yaeesh Siddiqi M. Glass A. D. (1998b). Dynamic interactions between root NH4+ influx and long-distance N translocation in rice: insights into feedback processes. Plant Cell Physiol. 39, 1287–1293. doi: 10.1093/oxfordjournals.pcp.a029332. PMID: 40388063

[B28] Kronzucker H. J. Siddiqi M. Y. Glass A. D. (1997). Conifer root discrimination against soil nitrate and the ecology of forest succession. Nature 385, 59–61. doi: 10.1038/385059a0. PMID: 37880705

[B29] Krouk G. Lacombe B. Bielach A. Perrine-Walker F. Malinska K. Mounier E. . (2010). Nitrate-regulated auxin transport by NRT1.1 defines a mechanism for nutrient sensing in plants. Dev. Cell 18, 927–937. doi: 10.1016/j.devcel.2010.05.008. PMID: 20627075

[B30] Ladha J. Bruijn F. Malik K. (1997). Introduction: Assessing opportunities for nitrogen fixation in rice – a frontier project. Plant Soil 194, 1–10. doi: 10.1023/A:1004264423436. PMID: 41886696

[B31] Lampayan R. M. Rejesus R. M. Singleton G. R. Bouman B. A. (2015). Adoption and economics of alternate wetting and drying water management for irrigated lowland rice. Field Crops Res. 170, 95–108. doi: 10.1016/j.fcr.2014.10.013. PMID: 38826717

[B32] Lee S. Marmagne A. Park J. Fabien C. Yim Y. Kim S.-J. . (2020). Concurrent activation of OsAMT1;2 and OsGOGAT1 in rice leads to enhanced nitrogen use efficiency under nitrogen limitation. Plant J. 103, 7–20. doi: 10.1111/tpj.14794. PMID: 32369636 PMC7383903

[B33] Li Y. L. Fan X. R. Shen Q. R. (2008). The relationship between rhizosphere nitrification and nitrogen-use efficiency in rice plants. Plant Cell Environ. 31, 73–85. doi: 10.1111/j.1365-3040.2007.01737.x. PMID: 17944815

[B34] Lian L. Lin Y. Wei Y. He W. Cai Q. Huang W. . (2021). PEPC of sugarcane regulated glutathione S-transferase and altered carbon–nitrogen metabolism under different N source concentrations in Oryza sativa. BMC Plant Biol. 21, 287. doi: 10.1186/s12870-021-03071-w. PMID: 34167489 PMC8223297

[B35] Lin W. Okon Y. Hardy R. W. (1983). Enhanced mineral uptake by Zea mays and Sorghum bicolor roots inoculated with Azospirillum brasilense. Appl. Environ. Microbiol. 45, 1775–1779. doi: 10.1128/aem.45.6.1775-1779.1983. PMID: 16346311 PMC242537

[B36] Liu K. H. Huang C. Y. Tsay Y. F. (1999). CHL1 is a dual-affinity nitrate transporter of Arabidopsis involved in multiple phases of nitrate uptake. Plant Cell 11, 865–874. doi: 10.1105/tpc.11.5.865. PMID: 10330471 PMC144217

[B37] López-Coria M. Hernández-Mendoza J. L. Sánchez-Nieto S. (2016). Trichoderma asperellum induces maize seedling growth by activating the plasma membrane H+-ATPase. Mol. Plant-Microbe Interact. 29, 797–806. doi: 10.1094/MPMI-07-16-0138-R. PMID: 27643387

[B38] Mantelin S. Desbrosses G. Larcher M. Tranbarger T. J. Cleyet-Marel J.-C. Touraine B. (2006). Nitrate-dependent control of root architecture and N nutrition are altered by a plant growth-promoting Phyllobacterium sp. Planta 223, 591–603. doi: 10.1007/s00425-005-0106-y. PMID: 16160849

[B39] Masakapalli S. K. Kruger N. J. Ratcliffe R. G. (2013). The metabolic flux phenotype of heterotrophic Arabidopsis cells reveals a complex response to changes in nitrogen supply. Plant J. 74, 569–582. doi: 10.1111/tpj.12142. PMID: 23406511

[B40] Matsunami M. Hayashi H. Tominaga Y. Nagamura Y. Murai-Hatano M. Ishikawa-Sakurai J. . (2018). Effective methods for practical application of gene expression analysis in field-grown rice roots. Plant Soil 433, 173–187. doi: 10.1007/s11104-018-3834-z. PMID: 30311153

[B41] Miller A. J. Cramer M. D. (2005). Root nitrogen acquisition and assimilation. Plant Soil 274, 1–36. doi: 10.1007/s11104-004-0965-1. PMID: 30311153

[B42] Muratore C. Espen L. Prinsi B. (2021). Nitrogen uptake in plants: The plasma membrane root transport systems from a physiological and proteomic perspective. Plants 10, 681. doi: 10.3390/plants10040681. PMID: 33916130 PMC8066207

[B43] Okamoto Y. Suzuki T. Sugiura D. Kiba T. Sakakibara H. Hachiya T. (2019). Shoot nitrate underlies a perception of nitrogen satiety to trigger local and systemic signaling cascades in Arabidopsis thaliana. Soil Sci. Plant Nutr. 65, 56–64. doi: 10.1080/00380768.2018.1537643. PMID: 37339054

[B44] Ota K. Yamamoto Y. (1989). Promotion of assimilation of ammonium ions by simultaneous application of nitrate and ammonium ions in radish plants. Plant Cell Physiol. 30, 365–371. doi: 10.1093/oxfordjournals.pcp.a077751. PMID: 40388063

[B45] Peng B. Kong H. Li Y. Wang L. Zhong M. Sun L. . (2014). OsAAP6 functions as an important regulator of grain protein content and nutritional quality in rice. Nat. Commun. 5, 4847. doi: 10.1038/ncomms5847. PMID: 25209128 PMC4175581

[B46] Pii Y. Mimmo T. Tomasi N. Terzano R. Cesco S. Crecchio C. (2015). Microbial interactions in the rhizosphere: beneficial influences of plant growth-promoting rhizobacteria on nutrient acquisition process. Biol. Fertil. Soils. 51, 403–415. doi: 10.1007/s00374-015-0996-1. PMID: 30311153

[B47] Plett D. Toubia J. Garnett T. Tester M. Kaiser B. N. Baumann U. (2010). Dichotomy in the NRT gene families of dicots and grass species. PloS One 5, e15289. doi: 10.1371/journal.pone.0015289. PMID: 21151904 PMC2997785

[B48] Ren P. Zhou B. Bi Y. Chen X. Yao S. Yang X. (2025). Bacillus subtilis can promote cotton phenotype, yield, nutrient uptake and water use efficiency under drought stress by optimizing rhizosphere microbial community in arid area. Ind. Crops Prod. 227, 120784. doi: 10.1016/j.indcrop.2025.120784. PMID: 38826717

[B49] Revsbech N. Pedersen O. Reichardt W. Briones A. (1999). Microsensor analysis of oxygen and pH in the rice rhizosphere under field and laboratory conditions. Biol. Fertil. Soils. 29, 379–385. doi: 10.1007/s003740050568. PMID: 30311153

[B50] Ruan J. Zhang F. Wong M. H. (2000). Effect of nitrogen form and phosphorus source on the growth, nutrient uptake and rhizosphere soil property of Camellia sinensis L. Plant Soil 223, 65–73. doi: 10.1023/A:1004882001803. PMID: 41886696

[B51] Sakakibara H. Takei K. Hirose N. (2006). Interactions between nitrogen and cytokinin in the regulation of metabolism and development. Trends Plant Sci. 11, 440–448. doi: 10.1016/j.tplants.2006.07.004. PMID: 16899391

[B52] Santamaria P. Elia A. (1997). Producing nitrate-free endive heads: effect of nitrogen form on growth, yield, and ion composition of endive. J. Amer. Soc Hortic. Sci. 122, 140–145. doi: 10.21273/JASHS.122.1.140

[B53] Scheible W. R. Gonzalez-Fontes A. Lauerer M. Muller-Rober B. Caboche M. Stitt M. (1997). Nitrate acts as a signal to induce organic acid metabolism and repress starch metabolism in tobacco. Plant Cell 9, 783–798. doi: 10.1105/tpc.9.5.783. PMID: 12237366 PMC156956

[B54] Shinjo R. Tanaka A. Sugiura D. Suzuki T. Uesaka K. Takebayashi Y. . (2020). Comprehensive analysis of the mechanisms underlying enhanced growth and root N acquisition in rice by the endophytic diazotroph, Burkholderia Vietnamiensis RS1. Plant Soil 450, 537–555. doi: 10.1007/s11104-020-04506-3. PMID: 30311153

[B55] Shinjo R. Uesaka K. Ihara K. Sakazaki S. Yano K. Kondo M. . (2018). Draft genome sequence of Burkholderia Vietnamiensis strain RS1, a nitrogen-fixing endophyte isolated from sweet potato. Microbiol. Resour. Announc. 7, e00820–18. doi: 10.1128/MRA.00820-18. PMID: 30533867 PMC6211351

[B56] Sweetlove L. J. Beard K. F. M. Nunes-Nesi A. Fernie A. R. Ratcliffe R. G. (2010). Not just a circle: flux modes in the plant TCA cycle. J. Exp. Bot. 61, 1437–1450. doi: 10.1093/jxb/erq056. PMID: 20554469

[B57] Tang S. Y. Hara S. Melling L. Goh K. J. Hashidoko Y. (2010). Burkholderia Vietnamiensis isolated from root tissues of nipa palm (Nypa fruticans) in Sarawak, Malaysia, proved to be its major endophytic nitrogen-fixing bacterium. Biosci. Biotechnol. Biochem. 74, 1972–1975. doi: 10.1271/bbb.100397. PMID: 20834139

[B58] Tran Van V. Berge O. Ke S. N. Balandreau J. Heulin T. (2000). Repeated beneficial effects of rice inoculation with a strain of Burkholderia Vietnamiensis on early and late yield components in low fertility sulphate acid soils of Vietnam. Plant Soil 218, 273–284. doi: 10.1023/A:1014986916913. PMID: 41886696

[B59] Wallner A. Busset N. Lachat J. Guigard L. King E. Rimbault I. . (2022). Differential genetic strategies of Burkholderia Vietnamiensis and Paraburkholderia kururiensis for root colonization of Oryza sativa subsp. japonica and O. sativa subsp. indica, as revealed by transposon mutagenesis sequencing. Appl. Environ. Microbiol. 88, e00642–22. doi: 10.1128/aem.00642-22. PMID: 35862731 PMC9317867

[B60] Wang M. Y. Siddiqi M. Y. Ruth T. J. Glass A. D. (1993). Ammonium uptake by rice roots (II. Kinetics of ^13^NH_4_^+^ influx across the plasmalemma). Plant Physiol. 103, 1259–1267. doi: 10.1104/pp.103.4.1259. PMID: 12232018 PMC159114

[B61] Wang W. Hu B. Yuan D. Liu Y. Che R. Hu Y. . (2018). Expression of the nitrate transporter gene OsNRT1.1A/OsNPF6.3 confers high yield and early maturation in rice. Plant Cell 30, 638–651. doi: 10.1105/tpc.17.00809. PMID: 29475937 PMC5894839

[B62] White P. J. (2003). “ Ion transport,” in Encyclopedia of Applied Plant Sciences. Eds. Thomas B. Murphy D. J. Murray B. G. ( Academic Press, Waltham, MA), 625–634.

[B63] Ying-Hua D. Ya-Li Z. Qi-Rong S. Song-Wei W. (2006). Nitrate effect on rice growth and nitrogen absorption and assimilation at different growth stages. Pedosphere 16, 707–717. doi: 10.1016/S1002-0160(06)60106-9

[B64] Youngdahl L. J. Pacheco R. Street J. J. Vlek P. L. G. (1982). The kinetics of ammonium and nitrate uptake by young rice plants. Plant Soil 69, 225–232. doi: 10.1007/BF02374517. PMID: 30311153

[B65] Yun C. A. O. Xiao-Rong F. A. N. Shu-Bin S. U. N. Guo-Hua X. U. Jiang H. U. Qi-Rong S. H. E. N. (2008). Effect of nitrate on activities and transcript levels of nitrate reductase and glutamine synthetase in rice. Pedosphere 18, 664–673. doi: 10.1016/S1002-0160(08)60061-2

