# Supplementary figures and images for: Preferential enhancement of nitrate utilization in rice by endophytic Burkholderia vietnamiensis RS1
Source: Front Plant Sci. 2026 May 12;17:1753845. doi: 10.3389/fpls.2026.1753845 (PMC13201235; doi:10.3389/fpls.2026.1753845)

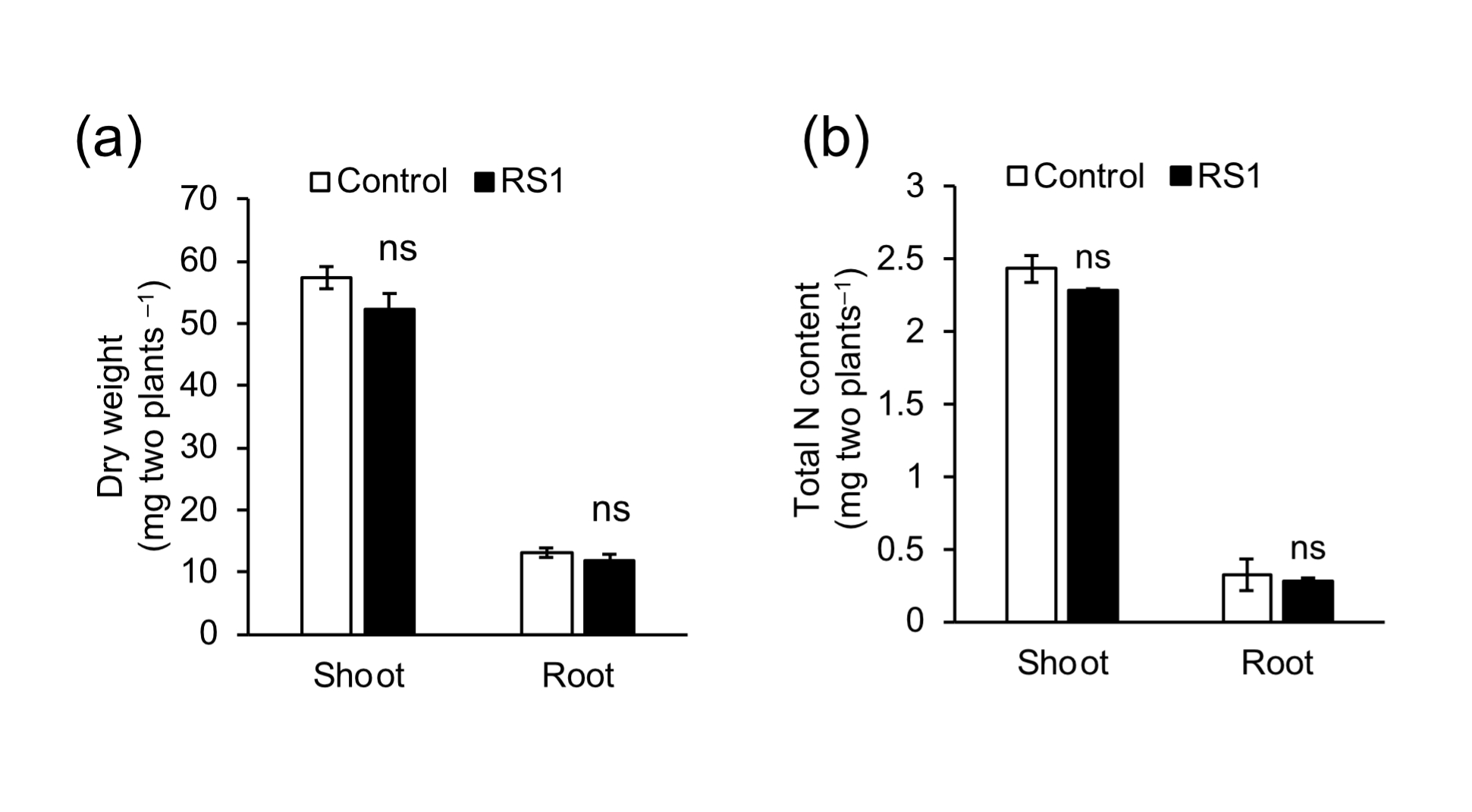

Supplement: Supplementary Figure 1 — Dry weight (a) and N content (b) of rice seedlings inoculated with B. vietnamiensis RS1 (RS1) or without inoculation (Control) at 15 days after sowing (DAS). Error bars indicate the standard error (n = 8). ns indicates no significant difference RS1 and Control (p < 0.05, Student t-test). [file Image1.jpeg]

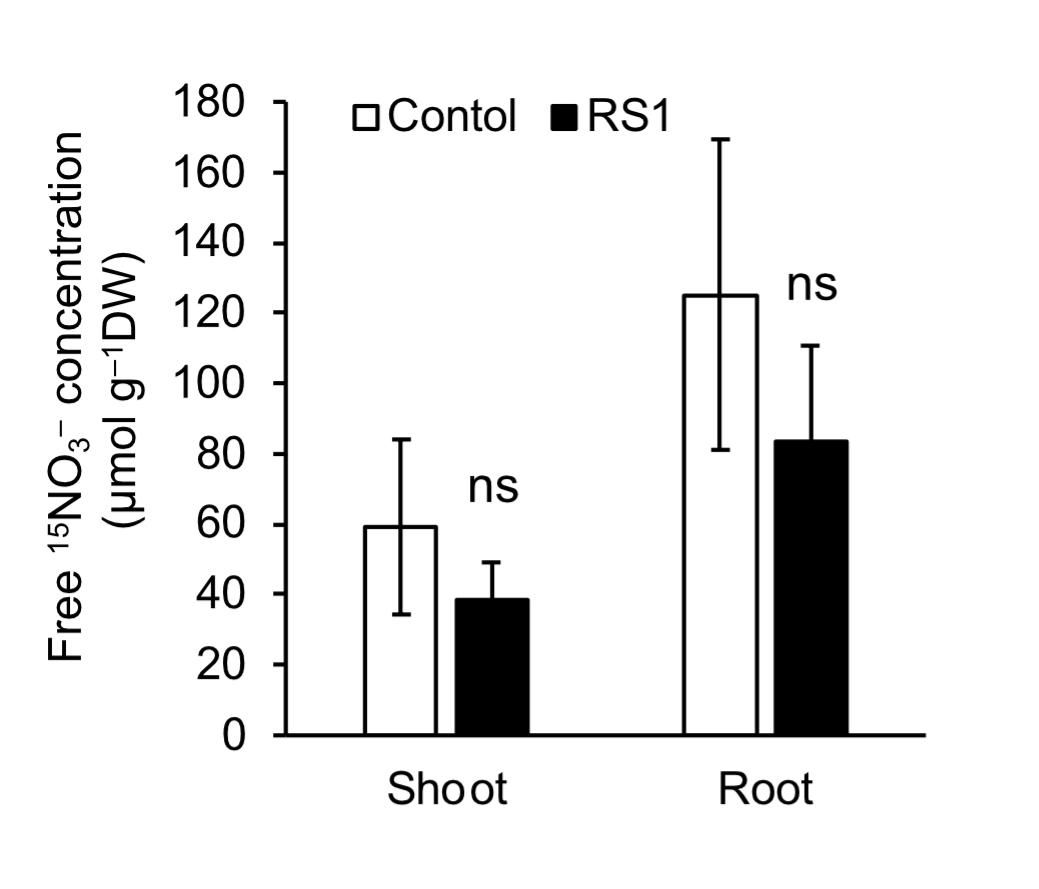

Supplement: Supplementary Figure 2 — Concentration of labeled free 15NO3– in rice seedlings supplied with 15NO3– at 15 days after sowing (DAS). Error bars indicate the standard error (n = 4). ns indicates no significant difference RS1 and Control (p < 0.05, Student t-test). [file Image2.jpeg]

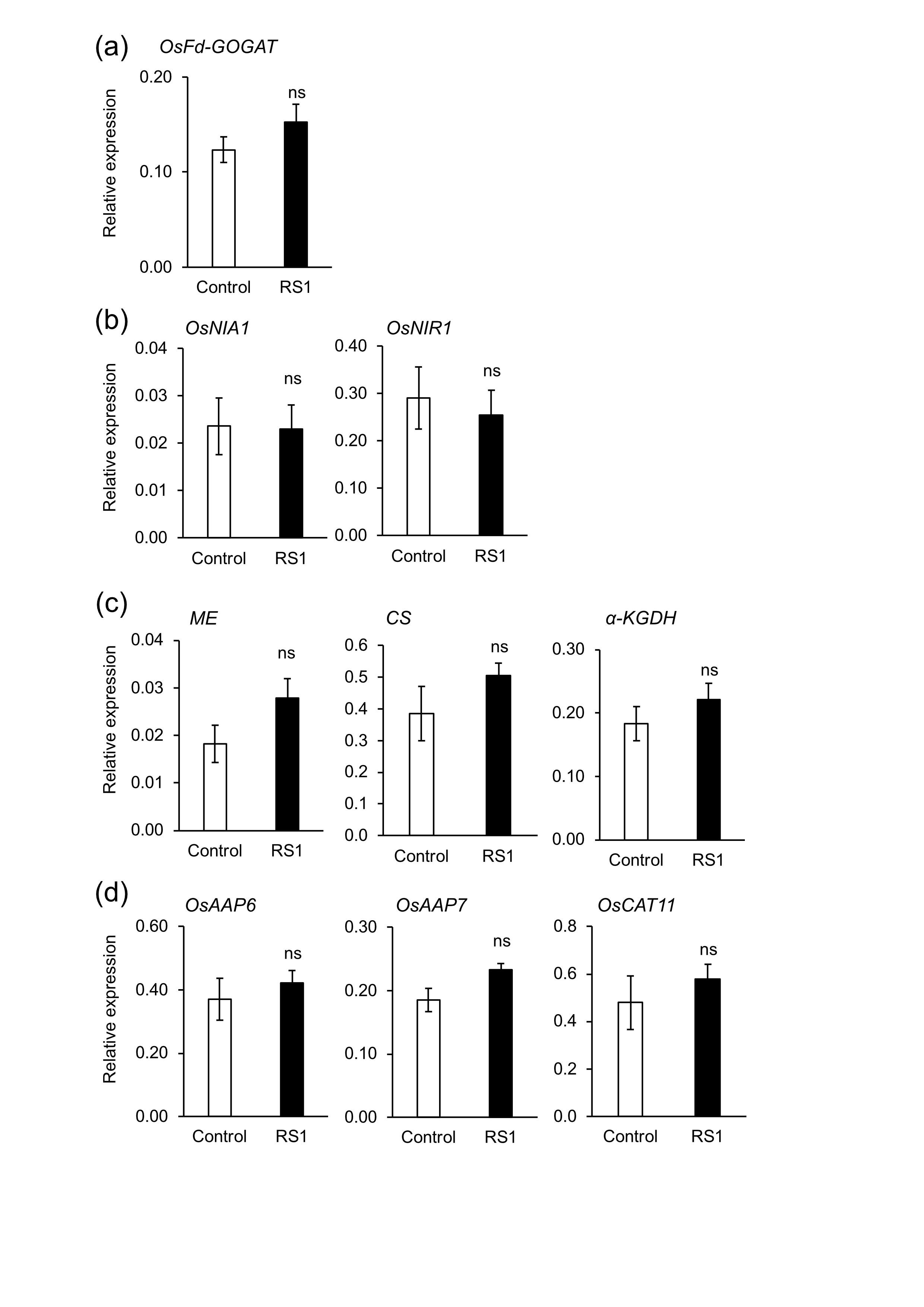

Supplement: Supplementary Figure 3 — RT-qPCR-based expression analyses of genes in roots of Control and RS1 at 21 days after sowing (DAS). (a) glutamate synthase (OsFd-GOGAT), (b) nitrate reductase (OsNIA1) and nitrite reductase (OsNIR1), (c) malic enzyme (ME), citrate synthase (CS), α-ketoglutarate dehydrogenase (α-KGDH), (d) amino acid transporters (OsAAP6, OsAAP7, OsCAT11). Error bars indicate the standard error (n = 3). ns indicates no significant difference RS1 and Control (p < 0.05, Student t-test). [file Image3.jpeg]

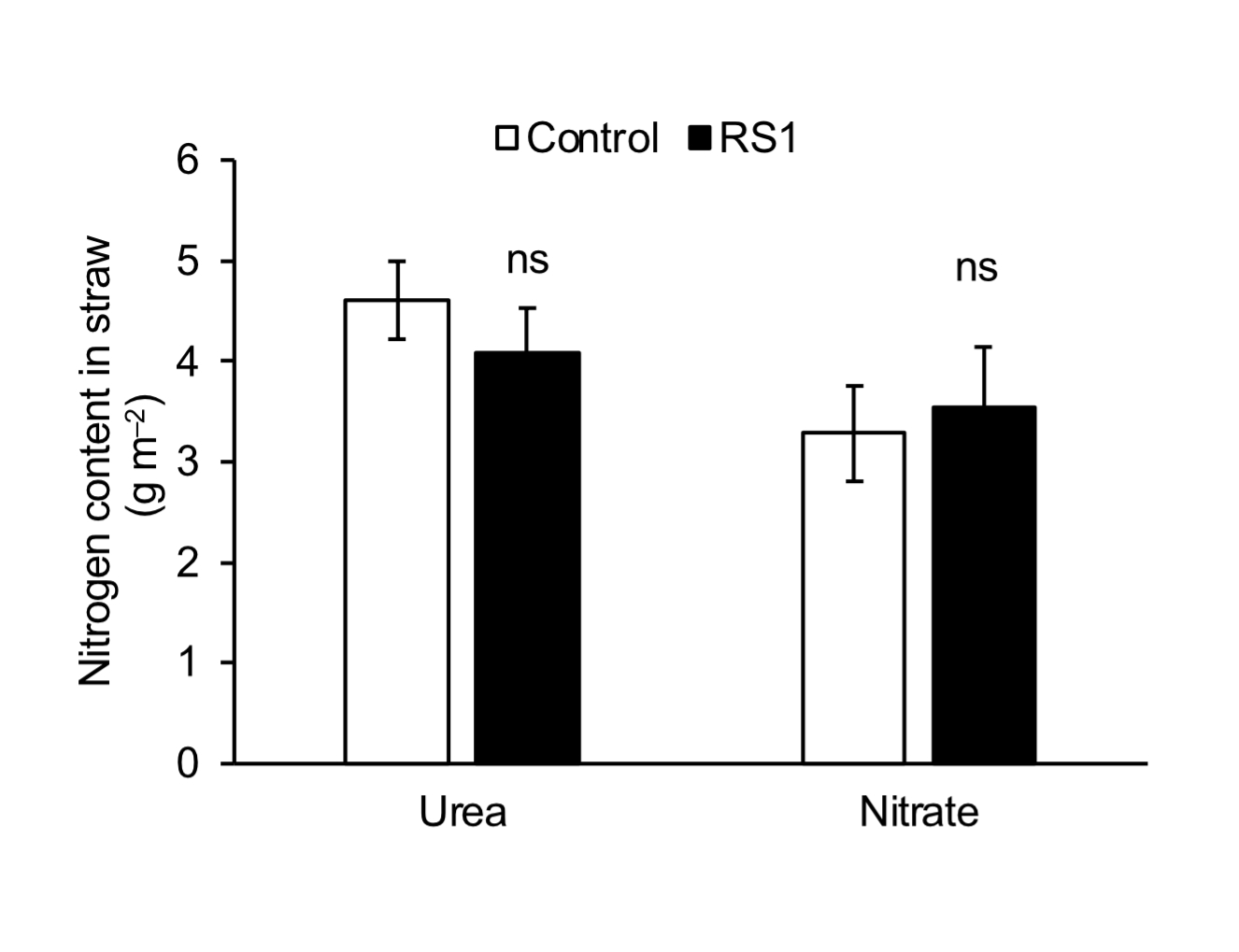

Supplement: Supplementary Figure 4 — Nitrogen content in the rice straw. Error bars indicate the standard error (n = 4). ns indicates no significant difference between RS1 and Control within each N fertilization condition. Statistical significance was evaluated separately for each N fertilization condition using linear mixed−effects models with block treated as a random effect (p < 0.05). [file Image4.jpeg]
