# Supplementary material for: Preferential enhancement of nitrate utilization in rice by endophytic Burkholderia vietnamiensis RS1
Source: Front Plant Sci. 2026 May 12;17:1753845. doi: 10.3389/fpls.2026.1753845 (PMC13201235; doi:10.3389/fpls.2026.1753845)
Supplement: Supplementary Table 1 — Primers used in this study. [file Table1.docx]

**Supplemental Table S1.** Primers used in this study.

|  |  |  |  |
| --- | --- | --- | --- |
| Gene | Forward sequence (5’-3’) | Reverse sequence (5’-3’) | Reference |
| *OsAMT1;1* | GGTCATCTTCGGGTGGGTCA | CGTGCCGTGTCAGGTCCAT | Duan et al., 2007 |
| *OsNRT1.1B* | GGCAGGCTCGACTACTTCTA | AGGCGCTTCTCCTTGTAGAC | Hu et al., 2015 |
| *OsNIA1* | GGAGGACGGGTGGGAGTA | TTCAGAAGACGAGGCAGGAC | Yun et al., 2008 |
| *OsNIR1* | CTGCCTCACCAAGGACAG | TTCCTACTCCTCGTCCTCCT | Wang et al., 2018 |
| *OsGS1.1* | CACCAACAAGAGGCACAATG | ACTCCCACTGTCCTGGCAT | Wang et al., 2018 |
| *OsGS1.2* | GTGATGTGTCAACGTTGGATTT | AAATCAAGAACATCCGTCCCTA | Lee et al., 2020 |
| *OsFd-GOGAT* | ATTTAGTGATGGAAGGACAGTAGGAGC | GCCAGTTTGTAGGTCAACCGTTATC | Lian et al., 2021 |
| *ME* | ACTTTGCCAACCACAATGCTTTT | AGTTCCACCGACCACCTTGAGCG | Lian et al., 2021 |
| *CS* | GCATCTGATCTTGATCTCAAGTCCC | CCAGTCATCCCTCTCATCCCACC | Lian et al., 2021 |
| *ICDHc* | GCGTCGACTTCAGACTTCACGAAGGC | GGCCATTCTCTGACTGGACTGCAATG | Lian et al., 2021 |
| *α-KGDH* | GGGCACGATAGACGACATCAAATAC | AAAGGTTTGATTACCCACGGCTATC | Lian et al., 2021 |
| *OsAAP6* | TCTTCCAAACGCAGCTCTGA | CACCAGCAACTAGTACGAGT | Peng et al,2014 |
| *OsAAP7* | ATCAAGCGGGCGAACTGC | ATCTCGATGAGGATGAGGGAGT | Jin et al., 2024 |
| *OsCAT11* | CTTATGGGGCGAACGGAATG | GGGGTATCTGGGTCCATAGC | this study |
