# Supplementary material for: Preferential enhancement of nitrate utilization in rice by endophytic Burkholderia vietnamiensis RS1
Source: Front Plant Sci. 2026 May 12;17:1753845. doi: 10.3389/fpls.2026.1753845 (PMC13201235; doi:10.3389/fpls.2026.1753845)
Supplement: Supplementary Table 2 — Physical and chemical properties of experimental paddy field soil. [file Table2.docx]

**Supplemental Table S2. Physical and chemical properties of experimental paddy field soil.**

|  |  |  |  |  |  |  |  |  |  |  |  |  |  |
| --- | --- | --- | --- | --- | --- | --- | --- | --- | --- | --- | --- | --- | --- |
| Site | Texture | pH | Total Carbon | Total Nitrogen | Available N | Available Phosphorus  (P₂O₅) | Exchangeable Potassium  (K₂O) | Exchangeable Calcium  (CaO) | Exchangeable Magnesium  (MgO) | Available Silica (SiO₂) | Phosphate Absorption Coefficient | Cation Exchange Capacity (CEC) | Free Iron Oxide (Fe₂O₃) |
|  |  |  | % | % | mg/100g | mg/100g | mg/100g | mg/100g | mg/100g | mg/100g | mg/100g | meq/100g | % |
| Togo | Sandy clay (SC) | 6.49 | 1.99 | 0.19 | 2.6 | 13 | 14 | 117 | 17 | 29 | 700 | 6.7 | 1.2 |
