# Supplementary material for: Preferential enhancement of nitrate utilization in rice by endophytic Burkholderia vietnamiensis RS1
Source: Front Plant Sci. 2026 May 12;17:1753845. doi: 10.3389/fpls.2026.1753845 (PMC13201235; doi:10.3389/fpls.2026.1753845)
Supplement: Supplementary Table 3 — Weather data at a site near the experimental paddy field during the experimental period in Nagoya, Aichi Prefecture, Japan. [file Table3.docx]

**Supplemental Table S3. Weather data at a site near the experimental paddy field during the experimental period in Nagoya, Aichi Prefecture, Japan.**

|  |  |  |  |  |  |  |  |  |
| --- | --- | --- | --- | --- | --- | --- | --- | --- |
|  |  | Precipitation (mm) | Temperature (℃) | | | Humidity (%) | | Global solar radiation (MJ m^–2^) |
| Year | Month | Total | Mean | Max. | Min. | Mean | Min. | Mean |
| 2021 | May. | 254 | 19.5 | 30.7 | 9.5 | 69 | 18 | 16.1 |
|  | Jun. | 137 | 23.4 | 32.4 | 15.6 | 72 | 28 | 17.4 |
|  | Jul. | 313 | 27.4 | 36.4 | 21.4 | 77 | 39 | 17.8 |
|  | Aug. | 347 | 27.8 | 37.8 | 22.4 | 76 | 26 | 15.9 |
|  | Sep. | 224 | 24.1 | 31.7 | 18.7 | 78 | 33 | 12.6 |
